# Early Prediction and Monitoring of Treatment Response in Gastrointestinal Stromal Tumors by Means of Imaging: A Systematic Review

**DOI:** 10.3390/diagnostics12112722

**Published:** 2022-11-07

**Authors:** Ylva. A. Weeda, Gijsbert M. Kalisvaart, Floris H. P. van Velden, Hans Gelderblom, Aart. J. van der Molen, Judith V. M. G. Bovee, Jos A. van der Hage, Willem Grootjans, Lioe-Fee de Geus-Oei

**Affiliations:** 1 Department of Radiology, Leiden University Medical Center, 2333 ZA Leiden, The Netherlands; 2 Department of Medical Oncology, Leiden University Medical Center, 2333 ZA Leiden, The Netherlands; 3 Department of Radiology, Section of Abdominal Radiology, Leiden University Medical Center, 2333 ZA Leiden, The Netherlands; 4 Department of Pathology, Leiden University Medical Center, 2333 ZA Leiden, The Netherlands; 5 Department of Surgical Oncology, Leiden University Medical Center, 2333 ZA Leiden, The Netherlands; 6 Biomedical Photonic Imaging Group, University of Twente, 7522 NB Enschede, The Netherlands; 7 Department of Radiation Science & Technology, Technical University of Delft, 2629 JB Delft, The Netherlands

**Keywords:** gastrointestinal stromal tumor, prediction, response monitoring, FDG-PET, radiomics, tomography, X-ray computed, personalized medicine

## Abstract

Gastrointestinal stromal tumors (GISTs) are rare mesenchymal neoplasms. Tyrosine kinase inhibitor (TKI) therapy is currently part of routine clinical practice for unresectable and metastatic disease. It is important to assess the efficacy of TKI treatment at an early stage to optimize therapy strategies and eliminate futile ineffective treatment, side effects and unnecessary costs. This systematic review provides an overview of the imaging features obtained from contrast-enhanced (CE)-CT and 2-deoxy-2-[^18^F]fluoro-D-glucose ([^18^F]FDG) PET/CT to predict and monitor TKI treatment response in GIST patients. PubMed, Web of Science, the Cochrane Library and Embase were systematically screened. Articles were considered eligible if quantitative outcome measures (area under the curve (AUC), correlations, sensitivity, specificity, accuracy) were used to evaluate the efficacy of imaging features for predicting and monitoring treatment response to various TKI treatments. The methodological quality of all articles was assessed using the Quality Assessment of Diagnostic Accuracy Studies, v2 (QUADAS-2) tool and modified versions of the Radiomics Quality Score (RQS). A total of 90 articles were included, of which 66 articles used baseline [^18^F]FDG-PET and CE-CT imaging features for response prediction. Generally, the presence of heterogeneous enhancement on baseline CE-CT imaging was considered predictive for high-risk GISTs, related to underlying neovascularization and necrosis of the tumor. The remaining articles discussed therapy monitoring. Clinically established imaging features, including changes in tumor size and density, were considered unfavorable monitoring criteria, leading to under- and overestimation of response. Furthermore, changes in glucose metabolism, as reflected by [^18^F]FDG-PET imaging features, preceded changes in tumor size and were more strongly correlated with tumor response. Although CE-CT and [^18^F]FDG-PET can aid in the prediction and monitoring in GIST patients, further research on cost-effectiveness is recommended.

## 1. Introduction

Gastrointestinal stromal tumors (GISTs) are rare mesenchymal neoplasms affecting the entire gastrointestinal tract and are presumed to originate from the interstitial cells of Cajal [1,2]. About 80–90% of GISTs harbor kinase-activating mutations in either receptor tyrosine kinase protein (KIT) or platelet-derived growth factor receptor α (PDGRF-α) [3,4]. Complete surgical excision remains the only curative treatment option for GIST patients. Since GISTs are generally insensitive to radio- and chemotherapy, non-surgical treatment is limited to tyrosine kinase inhibitor (TKI) therapy. This targeted molecular therapy is part of routine clinical practice for unresectable and metastatic disease [5,6].

Adjuvant TKI treatment is used in high-risk GISTs to improve survival [7]. Unfortunately, due to the varying aggressive nature of GISTs, about one-third of the patients will relapse within three years after surgery with curative-intent [8]. For localized disease, TKI treatment can be given to attain size reduction of the primary tumor and improve chances of complete resection while maintaining an acceptable risk of complications [9,10]. About 20–25% of patients do not benefit from the neoadjuvant TKI treatment, as no complete or partial response is observed [11,12]. The rarity and complex biological nature of this disease, makes it difficult to differentiate between good and poor responders. For example, GISTs harboring a KIT exon 11 mutation have a good response to TKI treatment, whereas the same treatment is less effective in tumors with KIT exon 9 mutations [13]. Additionally, progressive disease is common during long-term TKI treatment due to acquired treatment resistance [14,15].

In the era of personalized medicine, it is of utmost importance to evaluate the efficacy of TKI treatment at an early stage in order to optimize therapy strategies and protect patients from futile ineffective treatment, unnecessary side-effects and healthcare costs. Contrast-enhanced computed tomography (CE-CT) and 2-deoxy-2-[^18^F]fluoro-D-glucose ([^18^F]FDG) PET/CT are considered useful for diagnosis and response monitoring in GIST patients. The imaging modalities offer information on tumor morphology, perfusion characteristics, as well as tumor glucose metabolism [7]. However, optimal use of imaging for predicting and monitoring TKI treatment in patients with GIST is still a subject of debate. This systematic review aims to elucidate the added value of CE-CT and [^18^F]FDG PET/CT imaging in the *prediction of response* and *early response monitoring* of TKI treatment in localized and advanced GISTs.

## 2. Methods

### 2.1. Search Strategy

From 29 April 2022 to 24 June 2022, the databases of PubMed, Web of Science, the Cochrane Library and Embase were systematically screened using predefined search queries (Supplementary Materials). The following terms and their corresponding synonyms were included: “gastrointestinal stromal tumors”, “(neo)adjuvant”, “TKI treatment” and “FDG-PET” and “Tomography, X-ray Computed” imaging. The search queries are wide-ranging and seek to cover the aspect of both response monitoring and prediction by including ‘monitoring’ and ‘prediction models’ as well as ‘radiomics’ and ‘prognostics’. In addition to these search terms, other terms, such as ‘patient selection’ and ‘personalized medicine’, were also added, since these articles presumably covered the subject of TKI treatment evaluation and its efficacy in specific patient groups as well. The search strategy was implemented in consultation with an experienced research directorate, and access to the databases was granted by the Leiden University Medical Center.

### 2.2. Article Selection

Articles were screened and considered eligible for full-text assessment if the title or abstract mentioned (i) quantitative outcome measures to evaluate the efficacy of imaging features (ii) retrieved from CE-CT and/or [^18^F]FDG PET/CT imaging (iii) in predicting or monitoring (neo)adjuvant TKI treatment response (iv) in localized and advanced GISTs. Response monitoring is defined as the evaluation of disease over the course of treatment using multiple medical imaging time points. Predicting response, however, solely involves the use of baseline scans made prior to TKI treatment administration. Articles assessing the prognostic value of different clinical and imaging parameters (e.g., risk of recurrence and metastatic potential) that can guide TKI treatment duration or timing for specific patient groups were also included, since these findings may improve patient selection in the future. Exclusion criteria comprised non-English and non-human studies, reviews, guidelines, recommendations, editorials, conference papers and abstracts. Case reports and studies analyzing less than ten patients were also excluded. If the title and abstract did not contain sufficient information, full-text evaluation was used for judgement of relevance.

Subsequently, the articles were screened on full-text and excluded if they did not meet the previously mentioned inclusion criteria or if full-texts were not available. During this assessment, the focus was primarily on quantitative outcome measure(s) of studies. Outcome measures that were included in this analysis were correlations, associations, area under the curve (AUC), sensitivity, specificity and accuracy. 

Finally, the reference lists from included articles were screened to find additional articles on this topic. The articles were independently assessed by the first two authors (Y.A.W., G.M.K.) and in cases of discrepancy, consensus reading was performed to make a final decision that led to either inclusion or exclusion.

### 2.3. Quality Assessment

Articles using a radiomics pipeline were assessed through the radiomics quality score (RQS). The RQS is a scoring system that assigns points to a radiomics study based on specific criteria, where a maximum score of 36 points can be awarded. In this paper, the RQS is modified to focus on the methodological aspects of the included studies. The following criteria were omitted from the RQS, yielding a modified RQS (RQS_m_); ‘imaging at multiple time points’, ‘trial database registry’ and ‘multivariable analysis on non-radiomics features’, since they were considered less relevant for the quality of the obtained models [16]. The criteria from the RQS_m_ were also used to create a quality assessment tool to assess studies on non-radiomics prediction models and correlational research. Modifying the RQS_m_ for non-radiomics studies yielded the RQS_m,nonrad_. This RQS_m,nonrad_ had a maximum score of ten points (Supplementary Materials). Articles were considered high quality if they reached a score above 50%. To assess applicability concerns and the risk of bias in articles covering the topic of monitoring, the Quality Assessment of Diagnostic Accuracy Studies Tool, Version 2 (QUADAS-2) was applied [17]. Articles on response monitoring were considered to have a high risk of bias or applicability concerns if two or more of the domains were scored as ‘high’ or ‘unclear’. Subsequently these articles were scored as low-quality. 

Quality assessment was performed by the first author (Y.A.W.). The quality score was not considered as an exclusion criterion, as the authors considered it important to review all relevant evidence [17,18,19]. 

### 2.4. Data Analysis

The eligible studies were categorized based on their topic concerning either response prediction or therapy monitoring. From each study, detailed information on the publication year, first author, patient groups, type of TKI treatment and imaging technique(s), was obtained. The specific CE-CT and [^18^F]FDG-PET imaging features and their corresponding conclusions on efficacy, along with the attributed quality score, were briefly summarized. In the results section, only studies that were considered to be high-quality, were analyzed in depth by clarifying conclusions on clinical relevance, discrepancies in results and insights on biological correlates.

#### 2.4.1. Response Prediction

In response prediction, imaging features from baseline/diagnostic CE-CT and [^18^F]FDG-PET/CT are retrieved to predict responder status, prior to TKI administration. Articles on this topic were divided into five categories: mutational status, proliferative activity, risk stratification, radiological response and prognosis. These categories were considered important, as they all influence treatment strategies. Clinical genotyping is essential for clinical decision making regarding neoadjuvant therapy, since the sensitivity and resistance towards TKI treatment in GISTs is dependent on the mutational status. In addition, patients with a high-risk GIST (and thus high proliferative activity) receive adjuvant TKI treatment for a period of three years to eliminate remaining disease and reduce chances of relapse [7]. Predicting whether patients will have a radiological response or a good prognosis at baseline could also aid the development of a more personalized TKI treatment.

#### 2.4.2. Therapy Monitoring

In therapy monitoring, one uses the visual and quantitative differences between baseline and follow-up scans to determine treatment response. The efficacy of CE-CT and [^18^F]FDG-PET are first discussed separately, followed by a qualitative comparison between both imaging modalities. 

## 3. Results

### 3.1. Search Strategy and Article Selection

**Figure 1:**
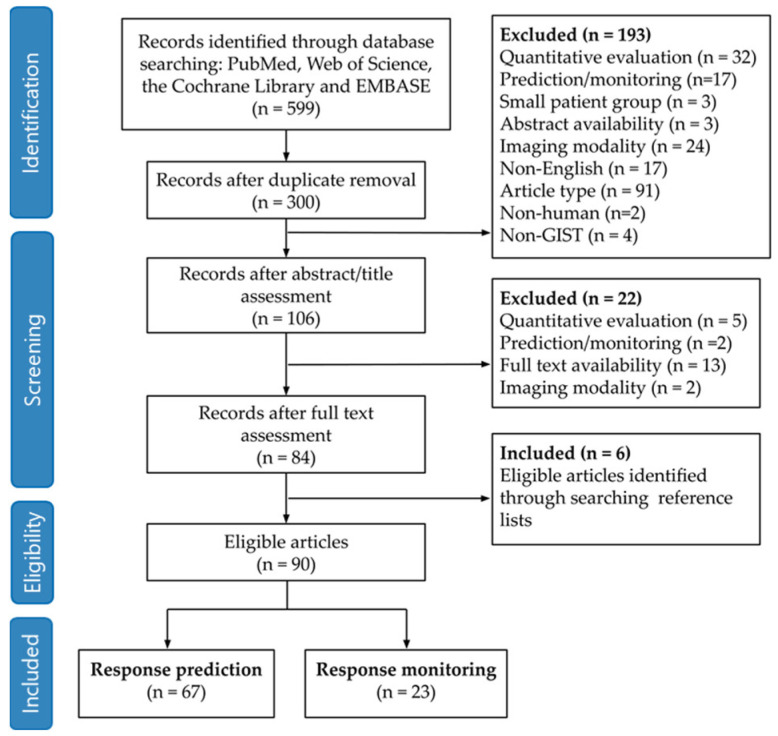
Preferred Reporting Items for Systematic Reviews and Meta-analysis (PRISMA) flowchart showing all the exclusion criteria. A total of 90 articles were included for this systematic review. Sixty-seven articles covered the topic of response prediction, and 23 articles covered the topic of response monitoring.

The search query identified a total of 599 articles from the databases of PubMed, Web of Science, the Cochrane library and Embase. The study selection process led to a total of 90 articles eligible for analysis (Figure 1). Articles that were excluded based on imaging criteria included, for example, the use of radiotracers other than [^18^F]FDG [20]. Additionally, some articles discussed the use of molecular genotyping and DNA sequencing to predict or determine response and therefore did not involve the use of any imaging modality [21]. Other excluded articles discussed the efficacy of a specific TKI treatment but did not quantify the efficacy of imaging features in predicting or monitoring response [22,23]. Of the 90 eligible articles, 67 were concerning response prediction [24,25,26,27,28,29,30,31,32,33,34,35,36,37,38,39,40,41,42,43,44,45,46,47,48,49,50,51,52,53,54,55,56,57,58,59,60,61,62,63,64,65,66,67,68,69,70,71,72,73,74,75,76,77,78,79,80,81,82,83,84,85,86,87,88,89,90] and 23 discussed response monitoring [91,92,93,94,95,96,97,98,99,100,101,102,103,104,105,106,107,108,109,110,111,112,113].

### 3.2. Quality Assessment

**Figure 2:**
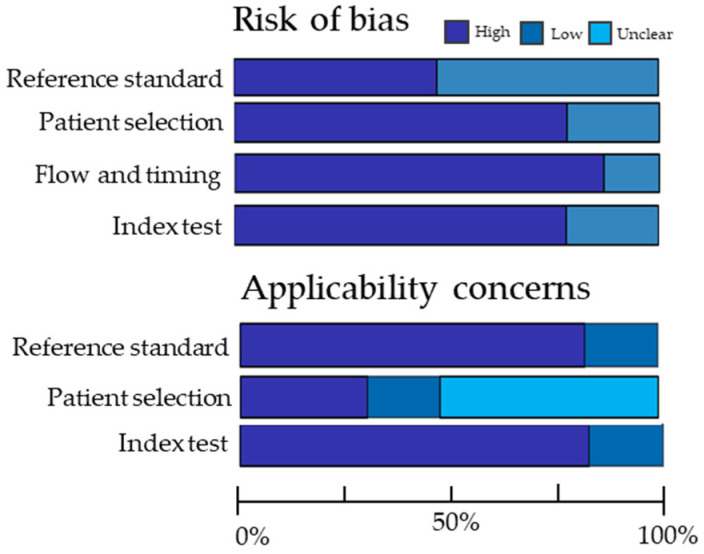
Summary of Methodological Quality Scored According to Quality Assessment of Diagnostic Accuracy Studies Tool, Version 2 (QUADAS-2) for 23 articles discussing the topic of response monitoring.

Twenty-two articles discussed the use of radiomic models, and six out of 22 studies were of low quality (score < 50%). The mean RQS_m_ of the included articles was 13.5 (SD ± 2.60) out of 26 points. Low scores were mainly caused by a lack of transparency, biological correlates and gold standard comparison. Two articles received a score of 18 points (69.2%), which was the highest attributed score [70,88]. The forty-five studies on non-radiomic prediction models and correlational research scored an average RQS_m,nonrad_ of 3.91 (SD ± 1.23) out of ten points, where eighteen articles scored above 50.0%. This was mainly caused by the fact that only a few articles used gold standard comparison [31,35,46] or an undescribed test set to validate their results [44,48,68]. The results of the QUADAS-2 tool are graphically displayed in Figure 2. Eight articles on response monitoring had high risk of bias or concerns for applicability and were therefore scored as low-quality. High risk of bias was often introduced by using reference standards involving follow-up (e.g., progression free survival, overall survival, time-to-treatment failure). Concerns for applicability were mainly caused by a lack of reporting on patient characteristics. In this way, judgement on whether the included patients matched the review question was unclear.

### 3.3. Response Prediction

All articles on response prediction have been summarized in the Supplementary Materials. Studies that were considered high-quality will be discussed in more detail.

#### 3.3.1. Mutational Status

The radiomic model of Starmans et al. was validated on unseen data and achieved an AUC, sensitivity and specificity of 0.51, 96.0% and 3.00% for predicting KIT mutation presence [81]. The model, based on portal venous radiomic features, requires further improvement in order to be clinically applicable. 

Three studies developed a model or nomogram based on radiomic features obtained from CE-CT imaging (arterial, venous and delayed phase) to predict the presence of KIT exon 11 mutations, which resulted in varying AUC outcomes, namely 0.57, 0.72 and 0.81 [75,76,81]. Deletions in exon 11 may indicate more aggressive tumor behavior, and for this reason, Liu et al. also assessed the efficacy of their model in predicting exon 11 deletion affecting codons 557–558 and achieved an AUC of 0.85 [76]. 

In clinical practice, patients with KIT exon 9 mutations often receive a high-dose imatinib regimen (800 mg) to improve progression-free survival (PFS). Yin et al. showed significantly greater tumor sizes and higher enhancement ratios (Hounsfield units (HU) for tumor parenchyma divided by the HUs of the erector spinae muscle) on portal venous CE-CT imaging compared to KIT exon 11 mutations. Using a 1.60 cut-off point, KIT exon 9 mutated small intestine tumors could be differentiated with an AUC, sensitivity and specificity of 0.76 and 86.7% and 98.5%, respectively. This threshold has, however, not been validated on independent validation data [67]. 

#### 3.3.2. Proliferative Activity

**Figure 3:**
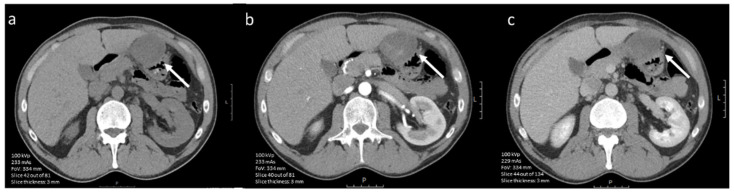
(**a**–**c**). Axial contrast-enhanced (iodinated contrast media) CT image of a 45-year-old male diagnosed with a (histopathologically confirmed) low mitotic gastric GIST. The lesion (arrow) shows a round tumor shape and homogeneous enhancement in (**a**) nonenhanced phase, (**b**) arterial phase and (**c**) portal venous phase (scale bars 5 cm).

**Figure 4:**
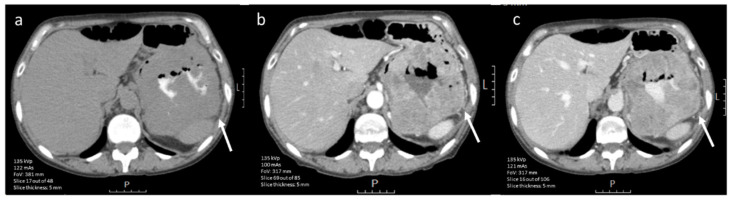
(**a**–**c**). Axial contrast-enhanced (iodinated contrast media) CT images of a 60-year-old male diagnosed with a (histopathologically confirmed) high-mitotic gastric GIST. The lesion (arrow) shows a lobulated tumor shape, heterogeneous enhancement in (**a**) nonenhanced phase, (**b**) arterial phase and (**c**) portal venous phase (scale bars 5 cm).

Since high-risk GISTs have a high proliferation rate, several studies attempted to link the mitotic index and Ki-67 proliferation index to imaging features in order to make a non-invasive assessment of expected tumor behavior. On CE-CT imaging, intralesional hypodensity and concurrent heterogeneous enhancement patterns were significantly more common in high-mitotic tumors (Figure 3 and Figure 4) [29,46]. Hypodensity was, in this case, defined as an area of low attenuation on portal venous phase CE-CT with HUs between 0 and 30 and when no HU increase (max 5 HUs) was observed between unenhanced and post-contrast images [46]. The changes in enhancement patterns were attributed to the principle of neovascularization. Tumors with high proliferative activity can induce the formation of hyperpermeable disorganized blood vessels and consequent development of necrosis [29,61]. Therefore, the supply and washout of contrast agent is affected, which has a direct impact on tumor enhancement patterns. 

A radiomic model using 42 quantitative and semantic imaging features (tumor location, first-order and texture radiomic features) retrieved from portal venous CE-CT imaging, differentiated high- from low-mitotic tumors with an AUC, sensitivity and specificity of 0.54, 27.0% and 75.0%, respectively [81]. Although on theoretical grounds CE-CT should be able to visualize poor neo-vasculature due to rapid tumor growth, no radiomic study has been able to establish this correlation. However, radiomic models predicting high Ki-67 proliferation index in localized and advanced GISTs achieved AUC values above 0.75 [77,88,89].

Comparison of studies investigating the relation between imaging and Ki-67 indices is complicated by the fact that different thresholds (e.g., 4%, 5%, 8% and 10%) for Ki-67 expression were used. Due to the small study sizes and heterogeneous outcomes with respect to Ki-67 indices, the true relationship between CE-CT imaging and proliferation has yet to be established.

#### 3.3.3. Risk Stratification

Research on the use of [^18^F]FDG-PET imaging features for risk stratification in GISTs is limited. In two studies, high metabolic tumor volume (MTV) and total lesion glycolysis (TLG) were predictive for high risk GISTs [25,35]. The use of quantitative imaging features showed improved predictive accuracy during follow-up when compared to a clinical reference standard (NIH modified criteria) [35]. Although these results suggest the added role of [^18^F]FDG-PET for risk stratification, there are only a few studies that investigated [^18^F]FDG-PET for this purpose. 

**Figure 5:**
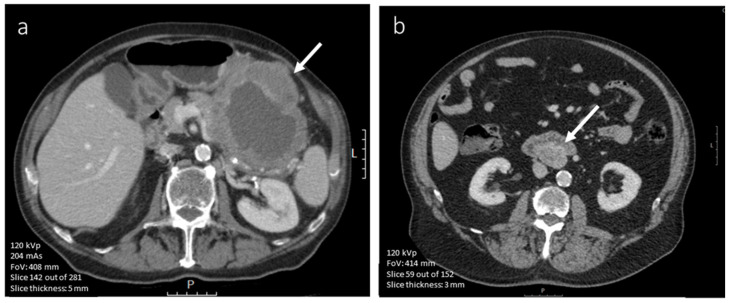
(**a**,**b**). Axial portal venous phase (iodinated contrast media) CT image of an 83-year-old male diagnosed with a high-risk (Miettinen AFIP classification) gastric GIST (scale bar 5 cm). The large lesion is lobulated and has central necrosis (arrow). (**b**) Axial portal venous phase (iodinated contrast media) CT slice of a 72-year-old male diagnosed with a low-risk GIST affecting the small intestine (scale bar 5 cm). It shows a well-defined and rounded lesion with a homogeneous enhancement pattern (arrow).

Larger tumor sizes, mixed or extra-luminal growth patterns, ill-defined tumor shape, presence of vessels feeding or vessels draining the tumor mass, necrosis and ulceration on CE-CT imaging were all associated with high-risk GISTs [44,53,58,60,63,64,68]. Of note, Wei et al. used the angle between the longest and shortest tumor diameter to quantify tumor shape. This parameter was able distinguish intermediate- and high-risk from low-risk GISTs more accurately when compared to using solely the longest diameter [58]. Heterogeneous enhancement patterns on portal venous phase CE-CT proved to be predictive for high-risk GISTs as well (Figure 5) [53]. Incomplete enhancement of the overlying gastric mucosa on arterial phase, was also significantly more common in high-risk gastric GISTs [51]. In a study by Tang et al., HUs of the arterial phase CE-CT were subtracted from the attenuation coefficients in the portal venous phase to derive quantitative features describing contrast enhancement. Using the subtraction CT, small regions of interest (ROIs) of 30–50 mm^2^, were placed in the most enhancing solid components of the tumors. The difference in HUs was significantly lower in high-risk gastric GISTs [53]. Additionally, the peak value of enhancement on CE-CT (arterial and portal venous phase) imaging was strongly correlated with risk [45]. Both articles suggest a rapid inflow of iodinated contrast agent in high-risk GISTs and thus the presence of permeable and leaky tumor vessels. The mean of the positive pixels (HU > 0) of the entire tumor volume on portal venous CT imaging was lower in high-risk GISTs [31]. This observation can be attributed to the presence of tumor necrosis, which was more commonly found in the high-risk group.

By contrast, Li et al. included gastric, intestinal and extra gastrointestinal tumors and did not find a significant difference in enhancement patterns between risk groups [43]. Although tumor enhancement has been established as a relevant factor in the risk stratification of GISTs, there are discrepancies in the results.

Machine learning used for the prediction of risk is extensively investigated with a total of twelve articles covering this topic [71,72,79,83,86,87]. All models achieved an AUC above 0.83 for predicting high-risk GISTs, with an average AUC of 0.87. In many of the models, texture radiomic features (gray level co-occurrence matrix (GLCM), neighboring gray-tone difference matrix (NGTDM) and gray run-length matrix (GRLM) and gray level size zoned matrix (GLSZM)) were used to develop the model. These texture features reflect enhancement patterns and inter-pixel relationships in a three-dimensional tumor volume. 

#### 3.3.4. Prediction of Radiological Response

There was one article attempting to predict radiological response using baseline imaging. Disease progression was in this case defined by the modified Choi criteria, which is currently one of the reference standards used for GIST response evaluation [114]. In this case, four textural portal venous features (features retrieved from GLCM, GLRLM and NGTDM) predicted disease progression with an AUC of 0.83 [32]. 

#### 3.3.5. Prognosis

Of the selected articles, two articles discussed the use of imaging features obtained from [^18^F]FDG-PET/CT imaging to predict PFS through detection of disease recurrence (locally and or development of distant metastases). They found significantly higher MTV and TLG values in patients with a lower PFS. In addition to quantitative [^18^F]FDG-PET imaging features, larger tumor sizes were also a significant factor contributing to lower PFS [25,35]. 

On CE-CT imaging, one study with a relatively large patient group (n = 143) observed that tumor sizes greater than 10 cm, ill-defined tumor outline and enhancing solid components contributed to a poor patient prognosis, as reflected by their overall survival (OS) [48]. The study by Jung et al. combined relevant predictive parameters (tumor location, ill-defined tumor outline and presence of feeding vessels) to create a nomogram. The nomogram was internally validated and achieved an AUC of 0.86 [37]. In addition to semantic CT features, Ekert et al. assessed the efficacy of four quantitative textural features from portal venous phase CT imaging to predict prognosis of GIST patients. This study found that high values for these texture features were all associated with poor PFS [32].

In another study, three-year recurrence-free survival (RFS) was predicted by a deep learning ResNet model based on features retrieved from arterial phase CT images. Results show that, using an internal validation cohort, a predictive model with an AUC of 0.91 was obtained [70]. Furthermore, Zheng et al. investigated whether the occurrence of liver metastasis in high risk GISTs could be predicted. They found that a model based on portal venous CT radiomic features reached an AUC and accuracy of 0.87 and 84.9% [90]. 

### 3.4. Therapy Monitoring

All articles on therapy monitoring have been summarized in the Supplementary Materials. Studies that were considered high-quality will be discussed in more detail.

#### 3.4.1. CE-CT Imaging

Many articles discussed the use of the well-established Response Evaluation Criteria in Solid Tumors (RECIST 1.1) to assess tumor response. RECIST 1.1 is a method in which the sum of the longest diameter of (a maximum of 5) target lesions is used to evaluate treatment response. The RECIST scoring system categorizes patients into four types of response, namely complete response (disappearance of all lesions), partial response (≥30% reduction of the sum of the target lesions (SLD)), progressive disease (≥20% increase of the SLD compared to the smallest SLD ever measured) and stable disease (neither progressive disease nor partial response) [115]. Nonetheless, substantial tumor shrinkage is often not observed during effective TKI treatment. Subtle and moderate changes in tumor size may be more accurately quantified by means of volumetric measurements. This is shown by Schiavon et al., who showed that size changes in GIST liver metastases larger than 20% were more frequently detected by volumetric measurements compared to the RECIST 1.1 criteria [110]. By using solely one-dimensional measurements, one presumes tumors remain spherical and that response occurs equally along three orthogonal axes during TKI treatment. However, liver metastasis in GIST patients showed significant changes in morphology over the course of imatinib treatment, which was better reflected by an ellipsoid volumetric approach [109]. 

**Figure 6:**
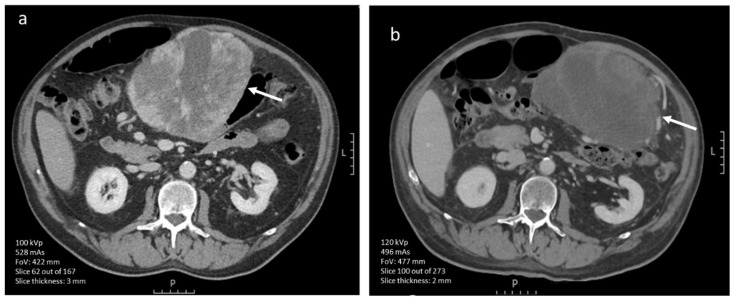
(**a**,**b**). Axial portal venous phase CT images (iodinated contrast media) of a 67-year-old male diagnosed with a primary GIST (arrows) of the stomach. (**a**) Pre-treatment imaging shows a large gastric mass with heterogeneous enhancement. (**b**) After 1.5 months of avapritinib treatment, the lesion has become hypodense (scale bars 5 cm).

In addition to RECIST 1.1, Choi et al. proposed a new method (Choi criteria) by including treatment-related changes in portal venous CT tumor densities [95]. Suppression of vascular endothelial growth factor expression can be induced by TKI treatment [116,117]. Therefore, treatment leads to changes in tumor vascularity and can lead to a reduction in tumor density, as reflected by the value of the HUs measured on CT (Figure 6). Using RECIST1.1 and Choi, comparable results were obtained for predicting PFS for patients treated with second line sunitinib assessed during an early follow-up of about 2–3 months [96,105]. Nonetheless, the Choi criteria gradually overestimated the number of patients with a partial response to sunitinib and regorafenib during longer follow-up periods (up to a year), leading to poorer PFS [105,106]. It was speculated that a drop in tumor density could also be caused by tumor necrosis, which is often a sign of progressive disease. So, instead of measuring a reduction in tumor vascularization, one may be measuring progressive disease over longer follow-up periods [105].

#### 3.4.2. [^18^F]FDG-PET Imaging

In [^18^F]FDG-PET imaging, the European Organization for Research and Treatment in Cancer (EORTC) PET criteria are most commonly used, in which a metabolic response is determined by a reduction in SUVmax of 25% or more [118]. Metabolic response was significantly associated with prolonged PFS and could be detected as early as seven days, after the induction of TKI treatment (imatinib and sunitinib) [100,102]. On the contrary, the prospective study of Chacón et al. did not find a significant association between PFS and metabolic response determined by the EORTC PET criteria.

Additionally, two retrospective studies by Farag et al. evaluated the impact of [^18^F]FDG-PET/CT on clinical decision making in the treatment of localized and advanced GIST patients. Changes in surgical management, systemic treatment and treatment objective were all included in the evaluation [111,112]. In 27.1% of GIST patients treated with neoadjuvant intent, management was changed because of [^18^F]FDG-PET/CT findings at an interval of eight weeks. The lack of metabolic response was correlated with therapeutic changes in management, especially in non-KIT exon 11 mutations [111]. In the advanced disease setting, specifically late [^18^F]FDG-PET response findings (median of 293 days) proved to have an impact on therapeutic decision [112]. 

#### 3.4.3. CE-CT vs. [^18^F]FDG-PET Imaging

**Figure 7:**
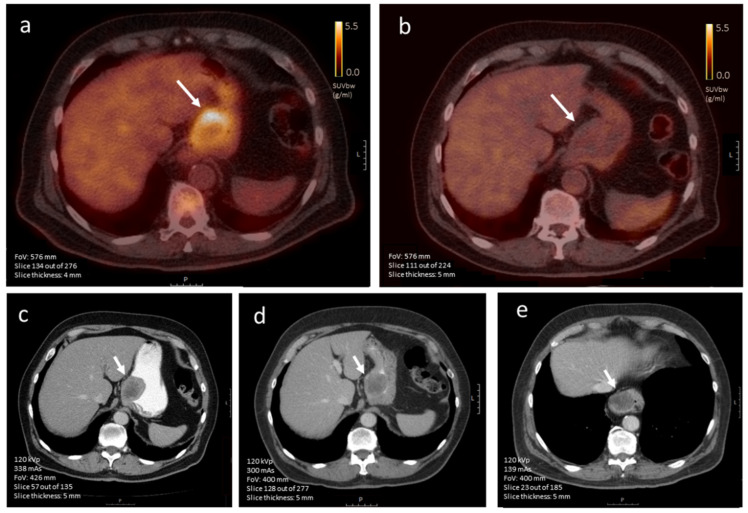
(**a**–**e**). Axial [^18^F]FDG-PET images of a 71-year-old male diagnosed with a primary gastric GIST (**a**) before treatment induction and (**b**) after about three months of TKI treatment, where the standardized uptake value (SUV) is normalized (scale bar 5 cm). Corresponding contrast-enhanced CT imaging (iodinated contrast media) visualizing the same lesion (arrow) (**c**) at diagnosis and after (**d**) 2.5 months and (**e**) 6.5 months of imatinib treatment, showing minimal to no change in tumor size (scale bar 5 cm). In the last image, the intrathoracic tumor location is caused by a sliding hernia.

When comparing the aforementioned response evaluation criteria on CE-CT imaging with the EORTC PET criteria on [^18^F]FDG-PET imaging, articles reported high agreement and RECIST responders also showed significant reductions in SUVmax [91,98,100,108]. Choi et al. showed greater sensitivity and specificity (97.0% and 100%) when compared to the EORTC PET criteria [95]. Metabolic response could, however, be observed within a week and preceded changes in tumor size and volume in localized and advanced GIST patients treated with imatinib (Figure 7) [92,97,100,107]. By using the RECIST criteria, the early effect of TKI treatment may be underestimated. For example, Choi et al. showed that 70% of the stable disease RECIST patients had an SUVmax reduction between 61 and 100% at the two-month follow-up [94]. 

## 4. Discussion

The aim of this review was to provide an overview of the value of CE-CT and [^18^F]FDG PET/CT imaging to predict and monitor TKI treatment response in GIST patients.

There is limited literature available on the use of baseline [^18^F]FDG-PET findings to predict tumor response. Although there are only a few studies available, generally imaging features, such as MTV and TLG, were correlated with more aggressive tumor behavior. On the contrary, there is more data available on the potential of CE-CT imaging features to predict treatment response. Results indicate that larger tumor sizes (>5 cm), ill-defined or lobulated tumor outline, mixed or exophytic growth patterns, the presence of (enlarged) and feeding vessels are associated with patient outcome. The presence of heterogeneous enhancement patterns was a recurring observation in high-risk GISTs. The hypodensities observed on CE-CT imaging were devoted to the biological phenomena of neovascularization and necrosis. It should be noted that the correlation between hypodensities on radiological imaging and actual pathological necrosis and neovascularization in GIST tumors is still disputable. 

Many articles discussed the use of radiomic and deep learning models for response prediction on baseline CE-CT imaging. High performance scores were stated for models predicting RFS and risk stratifications, while mutational status remained difficult to predict with variable AUC values. Radiomics offers the possibility to identify clinically relevant imaging features that would normally be imperceptible to the naked eye. For example, it has proven to be difficult to obtain a sufficient amount of tissue samples from biopsy material, which makes it difficult to determine the mutational status or a reliable mitotic count. Additionally, if the mitotic count is determined on postoperative surgical specimens, the results can be inaccurate due to the occasional administration of neoadjuvant TKI treatment. It would, therefore, be very helpful if imaging could provide additional information, other than tumor size. Nonetheless, the biological explanation behind the efficacy of radiomic features was often missing in the included articles. Before advanced and objective learning techniques can be introduced in clinical practice, they should be clinically relevant and biologically meaningful. It is recommended to further explore the prediction of actual radiological response using semantic or quantitative imaging features selected based upon tumor biology. 

The three evaluation methods currently used to monitor response in GIST patients, are the RECIST, Choi and EORTC PET criteria. The main disadvantage of the RECIST criteria is the one-dimensional nature of its measurements, presuming a spherical tumor shape throughout the entire course of TKI treatment. To overcome this limitation, an additional set of criteria was developed by Choi et al. involving CT densities. The Choi criteria are occasionally applied in clinical practice. However, its efficacy and prognostic value in determining response in GIST patients remains unclear. Supposedly, the antiangiogenic effect of TKI treatment would lead to a consequent reduction in HU values. As previously stated, necrosis and heterogeneous enhancement patterns at baseline were considered predictive for more aggressively behaving tumors. Using reductions in CT densities as a criterion for response monitoring may, therefore, be misleading, since it can reflect a decrease in angiogenesis induced by TKI treatment, as well as necrosis induced by aggressive tumor behavior. This hypothesis was supported by literature, since response evaluation using Choi criteria led to an overestimation in the number of partial responders at longer follow-up periods.

[^18^F]FDG-PET proved to be useful in the early monitoring of GISTs, since significant reductions in SUVmax could be observed within a week of TKI treatment and metabolic changes preceded morphological changes in size. However, this imaging technique is often not considered for early response monitoring in clinical practice because of higher costs. Since some of the targeted treatments are more expensive than PET-CT scans, further research should, therefore, be focused on the cost-effectiveness of [^18^F]FDG-PET imaging in the treatment of GISTs. 

Particularly, the combined use of different imaging modalities, also known as multimodality imaging, might provide more detailed information that can assist in making early image-guided treatment decisions. The use of such a multimodality imaging approach might be useful to gather as much information as possible on the biological behavior of GIST. However, currently, no literature is available on the specific use of combining these different imaging modalities for response prediction or monitoring.

## 5. Conclusions

In conclusion, imaging features obtained from CE-CT and [^18^F]FDG PET/CT imaging can aid in the development of a more personalized treatment of GIST patients by enabling early prediction and monitoring of TKI therapy response. Heterogeneous enhancement patterns on baseline CE-CT imaging were predictive for high-risk GISTs, reflecting neovascularization and necrosis.

For the purpose of response monitoring, current RECIST and Choi criteria are still lacking sensitivity and are prone to errors when predicting or monitoring treatment response. [^18^F]FDG-PET is a promising imaging technique that visualizes functional metabolic changes in GISTs, which precedes measurable changes in tumor size. Although promising, the true added value of [^18^F]FDG-PET remains elusive, and research on cost-effectiveness is warranted.

Radiomics is an emerging topic in medicine and shows potential for the prediction of RFS and risk stratifications in GISTs. However, future research should mainly focus on clinical utility, explainability and correlation with actual tumor biology.

## Data Availability

The datasets generated during and/or analyzed during the current study are available from the corresponding author upon reasonable request.

## Supplementary Materials

The following supporting information can be downloaded at: https://www.mdpi.com/article/10.3390/diagnostics12112722/s1.

## References

[1] Balachandran V.P., DeMatteo R.P. (2014). Gastrointestinal stromal tumors: Who should get imatinib and for how long?. Adv. Surg..

[2] Zappacosta R., Zappacosta B., Capanna S., D’Angelo C., Gatta D., Rosini S. (2012). GISTs: From the History to the Tailored Therapy, Gastrointestinal Stromal Tumor.

[3] Joensuu H., Hohenberger P., Corless C.L. (2013). Gastrointestinal stromal tumour. Lancet.

[4] Wu C.E., Tzen C.Y., Wang S.Y., Yeh C.N. (2019). Clinical Diagnosis of Gastrointestinal Stromal Tumor (GIST): From the Molecular Genetic Point of View. Cancers.

[5] Verweij J., Casali P.G., Zalcberg J., LeCesne A., Reichardt P., Blay J.Y., Issels R., van Oosterom A., Hogendoorn P.C., Van Glabbeke M. (2004). Progression-free survival in gastrointestinal stromal tumours with high-dose imatinib: Randomised trial. Lancet.

[6] Reichardt P. (2018). The Story of Imatinib in GIST—A Journey through the Development of a Targeted Therapy. Oncol. Res. Treat..

[7] Casali P.G., Blay J.Y., Abecassis N., Bajpai J., Bauer S., Biagini R., Bielack S., Bonvalot S., Boukovinas I., Bovee J. (2022). Gastrointestinal stromal tumours: ESMO-EURACAN-GENTURIS Clinical Practice Guidelines for diagnosis, treatment and follow-up. Ann. Oncol..

[8] Casali P.G., Le Cesne A., Poveda Velasco A., Kotasek D., Rutkowski P., Hohenberger P., Fumagalli E., Judson I.R., Italiano A., Gelderblom H. (2015). Time to Definitive Failure to the First Tyrosine Kinase Inhibitor in Localized GI Stromal Tumors Treated With Imatinib As an Adjuvant: A European Organisation for Research and Treatment of Cancer Soft Tissue and Bone Sarcoma Group Intergroup Randomized Trial in Collaboration with the Australasian Gastro-Intestinal Trials Group, UNICANCER, French Sarcoma Group, Italian Sarcoma Group, and Spanish Group for Research on Sarcomas. J. Clin. Oncol..

[9] Lopes L.F., Bacchi C.E. (2010). Imatinib treatment for gastrointestinal stromal tumour (GIST). J. Cell Mol. Med..

[10] Reynoso D., Trent J.C. (2010). Neoadjuvant and adjuvant imatinib treatment in gastrointestinal stromal tumor: Current status and recent developments. Curr. Opin. Oncol..

[11] Eisenberg B.L., Trent J.C. (2011). Adjuvant and neoadjuvant imatinib therapy: Current role in the management of gastrointestinal stromal tumors. Int. J. Cancer.

[12] Yang W., Liu Q., Lin G., Zhang B., Cao H., Zhao Y., Xia L., Feng F., Xiong Z., Hu J. (2021). The effect of neoadjuvant imatinib therapy on outcome and survival in rectal gastrointestinal stromal tumors: A multiinstitutional study. J. Surg. Oncol..

[13] Miettinen M., Lasota J. (2006). Gastrointestinal stromal tumors: Review on morphology, molecular pathology, prognosis, and differential diagnosis. Arch. Pathol. Lab. Med..

[14] Betz M., Kopp H.G., Spira D., Claussen C.D., Horger M. (2013). The benefit of using CT-perfusion imaging for reliable response monitoring in patients with gastrointestinal stromal tumor (GIST) undergoing treatment with novel targeted agents. Acta Radiol..

[15] Cassier P.A., Fumagalli E., Rutkowski P., Schöffski P., Van Glabbeke M., Debiec-Rychter M., Emile J.F., Duffaud F., Martin-Broto J., Landi B. (2012). Outcome of patients with platelet-derived growth factor receptor alpha-mutated gastrointestinal stromal tumors in the tyrosine kinase inhibitor era. Clin. Cancer Res..

[16] Lambin P., Leijenaar R.T.H., Deist T.M., Peerlings J., de Jong E.E.C., van Timmeren J., Sanduleanu S., Larue R., Even A.J.G., Jochems A. (2017). Radiomics: The bridge between medical imaging and personalized medicine. Nat. Rev. Clin. Oncol..

[17] Whiting P.F., Rutjes A.W., Westwood M.E., Mallett S., Deeks J.J., Reitsma J.B., Leeflang M.M., Sterne J.A., Bossuyt P.M. (2011). QUADAS-2: A revised tool for the quality assessment of diagnostic accuracy studies. Ann. Intern. Med..

[18] Jüni P., Witschi A., Bloch R., Egger M. (1999). The Hazards of Scoring the Quality of Clinical Trials for Meta-analysis. JAMA.

[19] Whiting P., Harbord R., Kleijnen J. (2005). No role for quality scores in systematic reviews of diagnostic accuracy studies. BMC Med. Res. Methodol..

[20] Pretze M., Reffert L., Diehl S., Schönberg S.O., Wängler C., Hohenberger P., Wängler B. (2021). GMP-compliant production of [(68)Ga]Ga-NeoB for positron emission tomography imaging of patients with gastrointestinal stromal tumor. EJNMMI Radiopharm. Chem..

[21] Hedenström P., Andersson C., Sjövall H., Enlund F., Nilsson O., Nilsson B., Sadik R. (2020). Pretreatment Tumor DNA Sequencing of KIT and PDGFRA in Endosonography-Guided Biopsies Optimizes the Preoperative Management of Gastrointestinal Stromal Tumors. Mol. Diagn. Ther..

[22] Cai P.Q., Lv X.F., Tian L., Luo Z.P., Mitteer R.A., Fan Y., Wu Y.P. (2015). CT Characterization of Duodenal Gastrointestinal Stromal Tumors. AJR Am. J. Roentgenol..

[23] Nannini M., Pantaleo M.A., Maleddu A., Saponara M., Mandrioli A., Lolli C., Pallotti M.C., Gatto L., Santini D., Paterini P. (2012). Duration of adjuvant treatment following radical resection of metastases from gastrointestinal stromal tumours. Oncol. Lett..

[24] Al-Balas H.A., Shaib Y.H. (2012). Gastrointestinal stromal tumours: Role of computed tomography in predicting tumour behaviour. Hong Kong J. Radiol..

[25] Albano D., Bosio G., Tomasini D., Bonù M., Giubbini R., Bertagna F. (2020). Metabolic behavior and prognostic role of pretreatment 18F-FDG PET/CT in gist. Asia Pac. J. Clin. Oncol..

[26] Cannella R., Tabone E., Porrello G., Cappello G., Gozzo C., Incorvaia L., Grignani G., Merlini A., D’Ambrosio L., Badalamenti G. (2021). Assessment of morphological CT imaging features for the prediction of risk stratification, mutations, and prognosis of gastrointestinal stromal tumors. Eur. Radiol..

[27] Chen T., Xu L., Dong X., Li Y., Yu J., Xiong W., Li G. (2019). The roles of CT and EUS in the preoperative evaluation of gastric gastrointestinal stromal tumors larger than 2 cm. Eur. Radiol..

[28] Chen X.S., Shan Y.C., Dong S.Y., Wang W.T., Yang Y.T., Liu L.H., Xu Z.H., Zeng M.S., Rao S.X. (2021). Utility of preoperative computed tomography features in predicting the Ki-67 labeling index of gastric gastrointestinal stromal tumors. Eur. J. Radiol..

[29] Chen Z., Yang J., Sun J., Wang P. (2020). Gastric gastrointestinal stromal tumours (2–5 cm): Correlation of CT features with malignancy and differential diagnosis. Eur. J. Radiol..

[30] Cho M.H., Park C.K., Park M., Kim W.K., Cho A., Kim H. (2015). Clinicopathologic Features and Molecular Characteristics of Glucose Metabolism Contributing to ¹⁸F-fluorodeoxyglucose Uptake in Gastrointestinal Stromal Tumors. PLoS ONE.

[31] Choi I.Y., Yeom S.K., Cha J., Cha S.H., Lee S.H., Chung H.H., Lee C.M., Choi J. (2019). Feasibility of using computed tomography texture analysis parameters as imaging biomarkers for predicting risk grade of gastrointestinal stromal tumors: Comparison with visual inspection. Abdom. Radiol..

[32] Ekert K., Hinterleitner C., Horger M. (2019). Prognosis assessment in metastatic gastrointestinal stromal tumors treated with tyrosine kinase inhibitors based on CT-texture analysis. Eur. J. Radiol..

[33] Fuster D., Ayuso J.R., Poveda A., Cubedo R., Casado A., Martinez-Trufero J., Lopez-Pousa A., Del Muro X.G., Lomena F., Maurel J. (2011). Value of FDG-PET for monitoring treatment response in patients with advanced GIST refractory to high-dose imatinib. A multicenter GEIS study. Q. J. Nucl. Med. Mol. Imaging.

[34] Grazzini G., Guerri S., Cozzi D., Danti G., Gasperoni S., Pradella S., Miele V. (2021). Gastrointestinal stromal tumors: Relationship between preoperative CT features and pathologic risk stratification. Tumori.

[35] Hwang S.H., Jung M., Jeong Y.H., Jo K., Kim S., Wang J., Cho A. (2021). Prognostic value of metabolic tumor volume and total lesion glycolysis on preoperative (18)F-FDG PET/CT in patients with localized primary gastrointestinal stromal tumors. Cancer Metab..

[36] Iannicelli E., Carbonetti F., Federici G.F., Martini I., Caterino S., Pilozzi E., Panzuto F., Briani C., David V. (2017). Evaluation of the Relationships between Computed Tomography Features, Pathological Findings, and Prognostic Risk Assessment in Gastrointestinal Stromal Tumors. J. Comput. Assist. Tomogr..

[37] Jung H., Lee S.M., Kim Y.C., Byun J., Park J.Y., Oh B.Y., Kwon M.J., Kim J. (2022). Gastrointestinal stromal tumours: Preoperative imaging features to predict recurrence after curative resection. Eur. J. Radiol..

[38] Kim H.C., Lee J.M., Kim K.W., Park S.H., Kim S.H., Lee J.Y., Han J.K., Choi B.I. (2004). Gastrointestinal stromal tumors of the stomach: CT findings and prediction of malignancy. AJR Am. J. Roentgenol..

[39] Kamiyama Y., Aihara R., Nakabayashi T., Mochiki E., Asao T., Kuwano H., Oriuchi N., Endo K. (2005). 18F-fluorodeoxyglucose positron emission tomography: Useful technique for predicting malignant potential of gastrointestinal stromal tumors. World J. Surg..

[40] Kim H.C., Lee J.M., Kim S.H., Park S.H., Lee J.W., Lee M., Han J.K., Choi B.I. (2005). Small gastrointestinal stromal tumours with focal areas of low attenuation on CT: Pathological correlation. Clin. Radiol..

[41] Kurata Y., Hayano K., Ohira G., Narushima K., Aoyagi T., Matsubara H. (2018). Fractal analysis of contrast-enhanced CT images for preoperative prediction of malignant potential of gastrointestinal stromal tumor. Abdom. Radiol..

[42] Kwon Y., Park E., Pahk K., Kim S., Kim M.J., Graf D., Park S. (2019). Preoperative assessment of malignant potential of gastrointestinal stromal tumor by dual-time-point 18F-fluorodeoxyglucose positron emission tomography imaging: Usefulness of standardized uptake value and retention index. J. Cancer Res. Ther..

[43] Li H., Ren G., Cai R., Chen J., Wu X., Zhao J. (2018). A correlation research of Ki67 index, CT features, and risk stratification in gastrointestinal stromal tumor. Cancer Med..

[44] Li C., Fu W., Huang L., Chen Y., Xiang P., Guan J., Sun C. (2021). A CT-based nomogram for predicting the malignant potential of primary gastric gastrointestinal stromal tumors preoperatively. Abdom. Radiol..

[45] Liu S., Pan X., Liu R., Zheng H., Chen L., Guan W., Wang H., Sun Y., Tang L., Guan Y. (2018). Texture analysis of CT images in predicting malignancy risk of gastrointestinal stromal tumours. Clin. Radiol..

[46] Mazzei M.A., Cioffi Squitieri N., Vindigni C., Guerrini S., Gentili F., Sadotti G., Mercuri P., Righi L., Lucii G., Mazzei F.G. (2020). Gastrointestinal stromal tumors (GIST): A proposal of a “CT-based predictive model of Miettinen index” in predicting the risk of malignancy. Abdom. Radiol..

[47] Miyake K.K., Nakamoto Y., Mikami Y., Tanaka S., Higashi T., Tadamura E., Saga T., Minami S., Togashi K. (2016). The predictive value of preoperative (18)F-fluorodeoxyglucose PET for postoperative recurrence in patients with localized primary gastrointestinal stromal tumour. Eur. Radiol..

[48] O’Neill A.C., Shinagare A.B., Kurra V., Tirumani S.H., Jagannathan J.P., Baheti A.D., Hornick J.L., George S., Ramaiya N.H. (2016). Assessment of metastatic risk of gastric GIST based on treatment-naïve CT features. Eur. J. Surg. Oncol..

[49] Pelandre G.L., Djahjah M.C., Gasparetto E.L., Nacif M.S., Marchiori E., De Mello E.L.R. (2013). To mographic findings of gastric gastrointestinal stromal tumor and correlation with the mitotic index. Arq. Gastroenterol..

[50] Palatresi D., Fedeli F., Danti G., Pasqualini E., Castiglione F., Messerini L., Massi D., Bettarini S., Tortoli P., Busoni S. (2022). Correlation of CT radiomic features for GISTs with pathological classification and molecular subtypes: Preliminary and monocentric experience. Radiol. Med..

[51] Peng G., Huang B., Yang X., Pang M., Li N. (2021). Preoperative CT feature of incomplete overlying enhancing mucosa as a high-risk predictor in gastrointestinal stromal tumors of the stomach. Eur. Radiol..

[52] Pinaikul S., Woodtichartpreecha P., Kanngurn S., Leelakiatpaiboon S. (2014). 1189 Gastrointestinal stromal tumor (GIST): Computed tomographic features and correlation of CT findings with histologic grade. J. Med. Assoc. Thai..

[53] Tang B., Feng Q.X., Liu X.S. (2022). Comparison of Computed Tomography Features of Gastric and Small Bowel Gastrointestinal Stromal Tumors with Different Risk Grades. J. Comput. Assist. Tomogr..

[54] Tateishi U., Hasegawa T., Satake M., Moriyama N. (2003). Gastrointestinal stromal tumor. Correlation of computed tomography findings with tumor grade and mortality. J. Comput. Assist. Tomogr..

[55] Tokumoto N., Tanabe K., Misumi T., Fujikuni N., Suzuki T., Ohdan H. (2014). The usefulness of preoperative 18FDG positron-emission tomography and computed tomography for predicting the malignant potential of gastrointestinal stromal tumors. Dig. Surg..

[56] Ulusan S., Koc Z., Kayaselcuk F. (2008). Gastrointestinal stromal tumours: CT findings. Br. J. Radiol..

[57] Verde F., Hruban R.H., Fishman E.K. (2017). Small Bowel Gastrointestinal Stromal Tumors: Multidetector Computed Tomography Enhancement Pattern and Risk of Progression. J. Comput. Assist. Tomogr..

[58] Wei S.C., Xu L., Li W.H., Li Y., Guo S.F., Sun X.R., Li W.W. (2020). Risk stratification in GIST: Shape quantification with CT is a predictive factor. Eur. Radiol..

[59] Xu D., Si G.Y., He Q.Z. (2020). Correlation analysis of multi-slice computed tomography (MSCT) findings, clinicopathological factors, and prognosis of gastric gastrointestinal stromal tumors. Transl. Cancer Res..

[60] Yang T.H., Hwang J.I., Yang M.S., Hung S.W., Chan S.W., Wang J., Tyan Y.S. (2007). Gastrointestinal stromal tumors: Computed tomographic features and prediction of malignant risk from computed tomographic imaging. J. Chin. Med. Assoc..

[61] Yang C.W., Liu X.J., Zhao L., Che F., Yin Y., Chen H.J., Zhang B., Wu M., Song B. (2021). Preoperative prediction of gastrointestinal stromal tumors with high Ki-67 proliferation index based on CT features. Ann. Transl. Med..

[62] Yoshikawa K., Shimada M., Kurita N., Sato H., Iwata T., Morimoto S., Miyatani T., Kashihara H., Takasu C., Matsumoto N. (2013). Efficacy of PET-CT for predicting the malignant potential of gastrointestinal stromal tumors. Surg. Today.

[63] Zhou C., Duan X., Zhang X., Hu H., Wang D., Shen J. (2016). Predictive features of CT for risk stratifications in patients with primary gastrointestinal stromal tumour. Eur. Radiol..

[64] Zhu M.P., Ding Q.L., Xu J.X., Jiang C.Y., Wang J., Wang C., Yu R.S. (2022). Building contrast-enhanced CT-based models for preoperatively predicting malignant potential and Ki67 expression of small intestine gastrointestinal stromal tumors (GISTs). Abdom. Radiol..

[65] Otomi Y., Otsuka H., Morita N., Terazawa K., Furutani K., Harada M., Nishitani H. (2010). Relationship between FDG uptake and the pathological risk category in gastrointestinal stromal tumors. J. Med. Investig..

[66] Park J.-W., Cho C.-H., Jeong D.-S., Chae H.-D. (2011). Role of F-fluoro-2-deoxyglucose Positron Emission Tomography in Gastric GIST: Predicting Malignant Potential Pre-operatively. J. Gastric Cancer.

[67] Yin Y.Q., Liu C.J., Zhang B., Wen Y., Yin Y. (2019). Association between CT imaging features and KIT mutations in small intestinal gastrointestinal stromal tumors. Sci. Rep..

[68] Wang J.K. (2017). Predictive value and modeling analysis of MSCT signs in gastrointestinal stromal tumors (GISTs) to pathological risk degree. Eur. Rev. Med. Pharmacol. Sci..

[69] Ao W., Cheng G., Lin B., Yang R., Liu X., Zhou C., Wang W., Fang Z., Tian F., Yang G. (2021). A novel CT-based radiomic nomogram for predicting the recurrence and metastasis of gastric stromal tumors. Am. J. Cancer Res..

[70] Chen T., Liu S., Li Y., Feng X., Xiong W., Zhao X., Yang Y., Zhang C., Hu Y., Chen H. (2019). Developed and validated a prognostic nomogram for recurrence-free survival after complete surgical resection of local primary gastrointestinal stromal tumors based on deep learning. EBioMedicine.

[71] Chen T., Ning Z., Xu L., Feng X., Han S., Roth H.R., Xiong W., Zhao X., Hu Y., Liu H. (2019). Radiomics nomogram for predicting the malignant potential of gastrointestinal stromal tumours preoperatively. Eur. Radiol..

[72] Chen Z.H., Xu L.Y., Zhang C.M., Huang C.C., Wang M.H., Feng Z., Xiong Y. (2021). CT Radiomics Model for Discriminating the Risk Stratification of Gastrointestinal Stromal Tumors: A Multi-Class Classification and Multi-Center Study. Front. Oncol..

[73] Chu H., Pang P., He J., Zhang D., Zhang M., Qiu Y., Li X., Lei P., Fan B., Xu R. (2021). Value of radiomics model based on enhanced computed tomography in risk grade prediction of gastrointestinal stromal tumors. Sci. Rep..

[74] Kang B., Yuan X., Wang H., Qin S., Song X., Yu X., Zhang S., Sun C., Zhou Q., Wei Y. (2021). Preoperative CT-Based Deep Learning Model for Predicting Risk Stratification in Patients With Gastrointestinal Stromal Tumors. Front. Oncol..

[75] Liu B., Liu H., Zhang L., Song Y., Yang S., Zheng Z., Zhao J., Hou F., Zhang J. (2022). Value of contrast-enhanced CT based radiomic machine learning algorithm in differentiating gastrointestinal stromal tumors with KIT exon 11 mutation: A two-center study. Diagn. Interv. Radiol..

[76] Liu X., Yin Y., Wang X., Yang C., Wan S., Yin X., Wu T., Chen H., Xu Z., Li X. (2021). Gastrointestinal stromal tumors: Associations between contrast-enhanced CT images and KIT exon 11 gene mutation. Ann. Transl. Med..

[77] Feng Q., Tang B., Zhang Y., Liu X. (2022). Prediction of the Ki-67 expression level and prognosis of gastrointestinal stromal tumors based on CT radiomics nomogram. Int. J. Comput. Assist. Radiol. Surg..

[78] Shao M., Niu Z., He L., Fang Z., He J., Xie Z., Cheng G., Wang J. (2021). Building Radiomics Models Based on Triple-Phase CT Images Combining Clinical Features for Discriminating the Risk Rating in Gastrointestinal Stromal Tumors. Front. Oncol..

[79] Ren C., Wang S., Zhang S. (2020). Development and validation of a nomogram based on CT images and 3D texture analysis for preoperative prediction of the malignant potential in gastrointestinal stromal tumors. Cancer Imaging.

[80] Ren C., Wang S., Zhang S., Jiang Z. (2019). Value of CT-Based Texture Analysis in Preoperative Prediction of the Grade of Gastrointestinal Stromal Tumors Compared to Conventional CT Imaging. Iran. J. Radiol..

[81] Starmans M.P.A., Timbergen M.J.M., Vos M., Renckens M., Grünhagen D.J., van Leenders G., Dwarkasing R.S., Willemssen F., Niessen W.J., Verhoef C. (2022). Differential Diagnosis and Molecular Stratification of Gastrointestinal Stromal Tumors on CT Images Using a Radiomics Approach. J. Digit. Imaging.

[82] Wang C., Li H., Jiaerken Y., Huang P., Sun L., Dong F., Huang Y., Dong D., Tian J., Zhang M. (2019). Building CT Radiomics-Based Models for Preoperatively Predicting Malignant Potential and Mitotic Count of Gastrointestinal Stromal Tumors. Transl. Oncol..

[83] Wang M., Feng Z., Zhou L., Zhang L., Hao X., Zhai J. (2021). Computed-Tomography-Based Radiomics Model for Predicting the Malignant Potential of Gastrointestinal Stromal Tumors Preoperatively: A Multi-Classifier and Multicenter Study. Front. Oncol..

[84] Xu F., Ma X., Wang Y., Tian Y., Tang W., Wang M., Wei R., Zhao X. (2018). CT texture analysis can be a potential tool to differentiate gastrointestinal stromal tumors without KIT exon 11 mutation. Eur. J. Radiol..

[85] Xu J., Zhou J., Wang X., Fan S., Huang X., Xie X., Yu R. (2020). A multi-class scoring system based on CT features for preoperative prediction in gastric gastrointestinal stromal tumors. Am. J. Cancer Res..

[86] Zhang L., Kang L., Li G., Zhang X., Ren J., Shi Z., Li J., Yu S. (2020). Computed tomography-based radiomics model for discriminating the risk stratification of gastrointestinal stromal tumors. Radiol. Med..

[87] Zhang Q.W., Zhou X.X., Zhang R.Y., Chen S.L., Liu Q., Wang J., Zhang Y., Lin J., Xu J.R., Gao Y.J. (2020). Comparison of malignancy-prediction efficiency between contrast and non-contract CT-based radiomics features in gastrointestinal stromal tumors: A multicenter study. Clin. Transl. Med..

[88] Zhang Q.W., Gao Y.J., Zhang R.Y., Zhou X.X., Chen S.L., Zhang Y., Liu Q., Xu J.R., Ge Z.Z. (2020). Personalized CT-based radiomics nomogram preoperative predicting Ki-67 expression in gastrointestinal stromal tumors: A multicenter development and validation cohort. Clin. Transl. Med..

[89] Zhao Y., Feng M., Wang M., Zhang L., Li M., Huang C. (2021). CT Radiomics for the Preoperative Prediction of Ki67 Index in Gastrointestinal Stromal Tumors: A Multi-Center Study. Front. Oncol..

[90] Zheng J., Xia Y., Xu A., Weng X., Wang X., Jiang H., Li Q., Li F. (2022). Combined model based on enhanced CT texture features in liver metastasis prediction of high-risk gastrointestinal stromal tumors. Abdom. Radiol..

[91] Antoch G., Kanja J., Bauer S., Kuehl H., Renzing-Koehler K., Schuette J., Bockisch A., Debatin J.F., Freudenberg L.S. (2004). Comparison of PET, CT, and dual-modality PET/CT imaging for monitoring of imatinib (STI571) therapy in patients with gastrointestinal stromal tumors. J. Nucl. Med..

[92] Beheshti M., Li S.R., Vali R., Schima W., Dudczak R., Langsteger W. (2007). The Potential Value of F-18 FDG PET in Comparison to CT in Early Prediction of Response to Imatinib (STI571) Therapy in Patients with Gastrointestinal Stromal Tumors. Iran. J. Nucl. Med..

[93] Chacón M., Eleta M., Espindola A.R., Roca E., Méndez G., Rojo S., Pupareli C. (2015). Assessment of early response to imatinib 800 mg after 400 mg progression by ¹⁸F-fluorodeoxyglucose PET in patients with metastatic gastrointestinal stromal tumors. Future Oncol..

[94] Choi H., Charnsangavej C., de Castro Faria S., Tamm E.P., Benjamin R.S., Johnson M.M., Macapinlac H.A., Podoloff D.A. (2004). CT evaluation of the response of gastrointestinal stromal tumors after imatinib mesylate treatment: A quantitative analysis correlated with FDG PET findings. AJR Am. J. Roentgenol..

[95] Choi H., Charnsangavej C., Faria S.C., Macapinlac H.A., Burgess M.A., Patel S.R., Chen L.L., Podoloff D.A., Benjamin R.S. (2007). Correlation of computed tomography and positron emission tomography in patients with metastatic gastrointestinal stromal tumor treated at a single institution with imatinib mesylate: Proposal of new computed tomography response criteria. J. Clin. Oncol..

[96] Dudeck O., Zeile M., Reichardt P., Pink D. (2011). Comparison of RECIST and Choi criteria for computed tomographic response evaluation in patients with advanced gastrointestinal stromal tumor treated with sunitinib. Ann. Oncol..

[97] Gayed I., Vu T., Iyer R., Johnson M., Macapinlac H., Swanston N., Podoloff D. (2004). The role of 18F-FDG PET in staging and early prediction of response to therapy of recurrent gastrointestinal stromal tumors. J. Nucl. Med..

[98] Goerres G.W., Stupp R., Barghouth G., Hany T.F., Pestalozzi B., Dizendorf E., Schnyder P., Luthi F., von Schulthess G.K., Leyvraz S. (2005). The value of PET, CT and in-line PET/CT in patients with gastrointestinal stromal tumours: Long-term outcome of treatment with imatinib mesylate. Eur. J. Nucl. Med. Mol. Imaging.

[99] Holdsworth C.H., Badawi R.D., Manola J.B., Kijewski M.F., Israel D.A., Demetri G.D., Van den Abbeele A.D. (2007). CT and PET: Early prognostic indicators of response to imatinib mesylate in patients with gastrointestinal stromal tumor. AJR Am. J. Roentgenol..

[100] Jager P.L., Gietema J.A., van der Graaf W.T. (2004). Imatinib mesylate for the treatment of gastrointestinal stromal tumours: Best monitored with FDG PET. Nucl. Med. Commun..

[101] Phongkitkarun S., Phaisanphrukkun C., Jatchavala J., Sirachainan E. (2008). Assessment of gastrointestinal stromal tumors with computed tomography following treatment with imatinib mesylate. World J. Gastroenterol..

[102] Prior J.O., Montemurro M., Orcurto M.V., Michielin O., Luthi F., Benhattar J., Guillou L., Elsig V., Stupp R., Delaloye A.B. (2009). Early prediction of response to sunitinib after imatinib failure by 18F-fluorodeoxyglucose positron emission tomography in patients with gastrointestinal stromal tumor. J. Clin. Oncol..

[103] Ryu M.H., Lee J.L., Chang H.M., Kim T.W., Kang H.J., Sohn H.J., Lee J.S., Kang Y.K. (2006). Patterns of progression in gastrointestinal stromal tumor treated with imatinib mesylate. Jpn. J. Clin. Oncol..

[104] Schindler E., Amantea M.A., Karlsson M.O., Friberg L.E. (2016). PK-PD modeling of individual lesion FDG-PET response to predict overall survival in patients with sunitinib-treated gastrointestinal stromal tumor. CPT Pharmacomet. Syst. Pharmacol..

[105] Schramm N., Englhart E., Schlemmer M., Hittinger M., Übleis C., Becker C.R., Reiser M.F., Berger F. (2013). Tumor response and clinical outcome in metastatic gastrointestinal stromal tumors under sunitinib therapy: Comparison of RECIST, Choi and volumetric criteria. Eur. J. Radiol..

[106] Shinagare A.B., Barysauskas C.M., Braschi-Amirfarzan M., O’Neill A.C., Catalano P.J., George S., Ramaiya N.H. (2016). Comparison of performance of various tumor response criteria in assessment of sunitinib activity in advanced gastrointestinal stromal tumors. Clin. Imaging.

[107] Van den Abbeele A.D., Gatsonis C., de Vries D.J., Melenevsky Y., Szot-Barnes A., Yap J.T., Godwin A.K., Rink L., Huang M., Blevins M. (2012). ACRIN 6665/RTOG 0132 phase II trial of neoadjuvant imatinib mesylate for operable malignant gastrointestinal stromal tumor: Monitoring with 18F-FDG PET and correlation with genotype and GLUT4 expression. J. Nucl. Med..

[108] Stroobants S., Goeminne J., Seegers M., Dimitrijevic S., Dupont P., Nuyts J., Martens M., van den Borne B., Cole P., Sciot R. (2003). 18FDG-Positron emission tomography for the early prediction of response in advanced soft tissue sarcoma treated with imatinib mesylate (Glivec). Eur. J. Cancer.

[109] Schiavon G., Ruggiero A., Bekers D.J., Barry P.A., Sleijfer S., Kloth J., Krestin G.P., Schöffski P., Verweij J., Mathijssen R.H. (2014). The effect of baseline morphology and its change during treatment on the accuracy of Response Evaluation Criteria in Solid Tumours in assessment of liver metastases. Eur. J. Cancer.

[110] Schiavon G., Ruggiero A., Schöffski P., van der Holt B., Bekers D.J., Eechoute K., Vandecaveye V., Krestin G.P., Verweij J., Sleijfer S. (2012). Tumor volume as an alternative response measurement for imatinib treated GIST patients. PLoS ONE.

[111] Farag S., Geus-Oei L.F., van der Graaf W.T., van Coevorden F., Grunhagen D., Reyners A.K.L., Boonstra P.A., Desar I., Gelderblom H., Steeghs N. (2018). Early Evaluation of Response Using (18)F-FDG PET Influences Management in Gastrointestinal Stromal Tumor Patients Treated with Neoadjuvant Imatinib. J. Nucl. Med..

[112] Farag S., NS I.J., Houdijk M.P.M., Reyners A.K.L., Arens A.I., Grünhagen D.J., Desar I.M.E., Gelderblom H., Steeghs N., de Geus-Oei L.F. (2021). Early response evaluation using 18F-FDG-PET/CT does not influence management of patients with metastatic gastrointestinal stromal tumors (GIST) treated with palliative intent. Nuklearmedizin.

[113] Goh B.K., Chow P.K., Chuah K.L., Yap W.M., Wong W.K. (2006). Pathologic, radiologic and PET scan response of gastrointestinal stromal tumors after neoadjuvant treatment with imatinib mesylate. Eur. J. Surg. Oncol..

[114] Arshad J., Ahmed J., Subhawong T., Trent J.C. (2020). Progress in determining response to treatment in gastrointestinal stromal tumor. Expert Rev. Anticancer Ther..

[115] Padhani A.R., Ollivier L. (2001). The RECIST (Response Evaluation Criteria in Solid Tumors) criteria: Implications for diagnostic radiologists. Br. J. Radiol..

[116] Faivre S., Demetri G., Sargent W., Raymond E. (2007). Molecular basis for sunitinib efficacy and future clinical development. Nat. Rev. Drug Discov..

[117] Jin T., Nakatani H., Taguchi T., Nakano T., Okabayashi T., Sugimoto T., Kobayashi M., Araki K. (2006). STI571 (Glivec) suppresses the expression of vascular endothelial growth factor in the gastrointestinal stromal tumor cell line, GIST-T1. World J. Gastroenterol..

[118] Young H., Baum R., Cremerius U., Herholz K., Hoekstra O., Lammertsma A.A., Pruim J., Price P. (1999). Measurement of clinical and subclinical tumour response using [^18^F]-fluorodeoxyglucose and positron emission tomography: Review and 1999 EORTC recommendations. European Organization for Research and Treatment of Cancer (EORTC) PET Study Group. Eur. J. Cancer.

