# Supplementary material for: Early Prediction and Monitoring of Treatment Response in Gastrointestinal Stromal Tumors by Means of Imaging: A Systematic Review
Source: Diagnostics (Basel). 2022 Nov 7;12(11):2722. doi: 10.3390/diagnostics12112722 (PMC9689665; doi:10.3390/diagnostics12112722)
Supplement: Supplementary file 1 [file diagnostics-12-02722-s001.zip › diagnostics-1999957-supplementary.pdf]

Supplementary materials: Search strings

Systematic review on CE-CT and 18F-FDG PET/CT imaging in the early prediction and monitoring of TKI treatment response in localised and advanced GISTs.

PubMed

((("Gastrointestinal Stromal Tumors"[mesh] OR "GIST tumor"[tw] OR "GIST tumors"[tw] OR "GIST tumour"[tw] OR "GIST tumours"[tw] OR "gastrointestinal tract stromal tumor"[tw] OR "gastrointestinal tract stromal tumors"[tw] OR "gastrointestinal stromal tumor"[tw] OR "gastrointestinal stromal tumors"[tw] OR "gastrointestinal stromal tumour"[tw] OR "gastrointestinal stromal tumours"[tw] OR "gastro intestinal stromal tumor"[tw] OR "gastro intestinal stromal tumors"[tw] OR "gastro intestinal stromal tumour"[tw] OR "gastro intestinal stromal tumours"[tw] OR (("gastrointestinal tract"[tw] OR "GI Tract"[tw] OR "GI"[tiab] OR "gastrointestinal"[tw] OR "gastro intestinal"[tw] OR "intestinal"[tw] OR "intestine"[tw] OR "intestines"[tw] OR "colon"[tw] OR "rectum"[tw] OR "anus"[tw] OR "colonic"[tw] OR "rectal"[tw] OR "anal"[tw]) AND ("stromal tumor"[tw] OR "stromal tumors"[tw] OR "stromal tumour"[tw] OR "stromal tumours"[tw] OR "stromal neoplasm"[tw] OR "stromal neoplasms"[tw] OR "stromal cancer"[tw] OR "stromal"[tw]))) AND ("neo adjuvant imatinib"[tw] OR "neoadjuvant imatinib"[tw] OR "neo adjuvant imatinib*"[tw] OR "neoadjuvant imatinib*"[tw] OR (("Imatinib Mesylate"[Mesh] OR "Imatinib"[tw] OR "Imatinib*"[tw] OR "Gleevec"[tw] OR "Glivec"[tw] OR "multitargeted tyrosine kinase inhibitors"[tw] OR "multitargeted tyrosine kinase inhibitor"[tw] OR "multi targeted tyrosine kinase inhibitors"[tw] OR "multi targeted tyrosine kinase inhibitor"[tw] OR "tyrosine kinase inhibitors"[tw] OR "tyrosine kinase inhibitor"[tw] OR "Protein-Tyrosine Kinases/antagonists and inhibitors"[Mesh]) AND ("Neoadjuvant Therapy"[Mesh] OR "Neoadjuvant"[tw] OR "Neoadjuvan*"[tw] OR "Neo adjuvant"[tw] OR "Neo adjuvan*"[tw] OR "Chemotherapy, Adjuvant"[Mesh] OR "adjuvant"[tw]))) AND ("Tomography, X-Ray Computed"[mesh] OR "CT"[tw] OR "computed tomography"[tw] OR "computer tomography"[tw] OR "computed tomogr*"[tw] OR "computer tomogr*"[tw] OR "Positron-Emission Tomography"[mesh] OR "Positron-Emission Tomography"[tw] OR "Positron Emission Tomogr*"[tw] OR "Positron-Emission Tomogr*"[tw] OR "PET"[tw]) AND ("Patient Selection"[Mesh] OR "Patient Selection"[tw] OR "Patients Selection"[tw] OR "Subject Selection"[tw] OR "Subjects Selection"[tw] OR "Patient Recruitment"[tw] OR "Patients Recruitment"[tw] OR "Subject Recruitment"[tw] OR "Subjects Recruitment"[tw] OR "selection"[tw] OR ″response evaluation″[tw] OR ″evaluate response″[tw] OR "select*"[tw] OR "recruitment"[tw] OR "recruit*"[tw] OR "Precision Medicine"[Mesh] OR "personalized medicine"[tw] OR "personalised medicine"[tw] OR "personalized"[tw] OR "personalised"[tw] OR "prediction models"[tw] OR "prediction model"[tw] OR "prediction"[tw] OR ″predict*″[tw] OR "monitoring"[tw] OR "monitor"[tw] OR "monitor*"[tw] OR "genomics"[tw] OR "genomic"[tw] OR "radiomics"[tw] OR "genomic*"[tw] OR "radiomic"[tw] OR "radiomic*"[tw] OR "machine learning"[tw] OR "Artificial Intelligence"[tw] OR "Machine Learning"[tw] OR "Deep Learning"[tw] OR "prognostic"[tw] OR ″prognostics″[tw] OR ″prognost*″[tw] OR "prognostic factors"[tw] OR "Imaging Genomics"[Mesh] OR "Genomics"[Mesh] OR "Artificial Intelligence"[mesh]))
OR (("Gastrointestinal Stromal Tumors"[majr] OR "GIST tumor"[ti] OR "GIST tumors"[ti] OR "GIST tumour"[ti] OR "GIST tumours"[ti] OR "gastrointestinal tract stromal tumor"[ti] OR "gastrointestinal tract stromal tumors"[ti] OR "gastrointestinal stromal tumor"[ti] OR "gastrointestinal stromal tumors"[ti] OR "gastrointestinal stromal tumour"[ti] OR "gastrointestinal stromal tumours"[ti] OR "gastro intestinal stromal tumor"[ti] OR "gastro intestinal stromal tumors"[ti] OR "gastro intestinal stromal tumour"[ti] OR "gastro intestinal stromal tumours"[ti] OR (("gastrointestinal tract"[ti] OR "GI Tract"[ti] OR "GI"[tiab] OR "gastrointestinal"[ti] OR "gastro intestinal"[ti] OR "intestinal"[ti] OR "intestine"[ti] OR "intestines"[ti] OR "colon"[ti] OR "rectum"[ti] OR "anus"[ti] OR "colonic"[ti] OR "rectal"[ti] OR "anal"[ti]) AND ("stromal tumor"[ti] OR "stromal tumors"[ti] OR "stromal tumour"[ti] OR "stromal tumours"[ti] OR "stromal neoplasm"[ti] OR "stromal neoplasms"[ti] OR "stromal cancer"[ti] OR "stromal"[ti]))) AND ("Patient Selection"[Mesh] OR "Patient Selection"[tw] OR "Patients Selection"[tw] OR "Subject Selection"[tw] OR "Subjects Selection"[tw] OR "Patient Recruitment"[tw] OR "Patients Recruitment"[tw] OR "Subject Recruitment"[tw] OR "Subjects Recruitment"[tw] OR "selection"[tw] OR ″response evaluation″[tw] OR ″evaluate response″[tw] OR "select*"[tw] OR "recruitment"[tw] OR "recruit*"[tw] OR "Precision Medicine"[Mesh] OR "personalized medicine"[tw] OR "personalised medicine"[tw] OR "personalized"[tw] OR "personalised"[tw] OR "prediction models"[tw] OR "prediction model"[tw] OR "prediction"[tw] OR ″predict*″[tw] OR "monitoring"[tw] OR "monitor"[tw] OR "monitor*"[tw] OR "genomics"[tw] OR "genomic"[tw] OR "radiomics"[tw] OR "genomic*"[tw] OR "radiomic"[tw] OR "radiomic*"[tw] OR "machine learning"[tw] OR "Artificial Intelligence"[tw] OR "Machine Learning"[tw] OR "Deep Learning"[tw] OR "prognostic"[tw] OR ″prognostics″[tw] OR ″prognost*″[tw] OR "prognostic factor"[tw] OR

"prognostic factors"[tw] OR "Imaging Genomics"[Mesh] OR "Genomics"[Mesh] OR "Artificial Intelligence"[mesh]) AND ("Tomography, X-Ray Computed"[majr] OR "CT"[ti] OR "computed tomography"[ti] OR "computed tomography"[ti] OR "computed tomogr*"[ti] OR "computer tomogr*"[ti] OR "Positron-Emission Tomography"[majr] OR "Positron-Emission Tomography"[ti] OR "Positron Emission Tomogr*"[tw] OR "Positron-Emission Tomogr*"[ti] OR "PET"[ti]))) NOT (("Case Reports"[ptyp] OR "case report"[ti]) NOT ("Review"[ptyp] OR "review"[ti] OR "Clinical Study"[ptyp] OR "trial"[ti] OR "RCT"[ti]))

Embase

(((exp *"Gastrointestinal Stromal Tumor"/ OR "GIST tumor".ti,ab OR "GIST tumors".ti,ab OR "GIST tumour".ti,ab OR "GIST tumours".ti,ab OR "gastrointestinal tract stromal tumor".ti,ab OR "gastrointestinal tract stromal tumors".ti,ab OR "gastrointestinal stromal tumor".ti,ab OR "gastrointestinal stromal tumors".ti,ab OR "gastrointestinal stromal tumour".ti,ab OR "gastrointestinal stromal tumours".ti,ab OR "gastro intestinal stromal tumor".ti,ab OR "gastro intestinal stromal tumors".ti,ab OR "gastro intestinal stromal tumour".ti,ab OR "gastro intestinal stromal tumours".ti,ab OR (("gastrointestinal tract".ti,ab OR "GI Tract".ti,ab OR "GI".ti,ab OR "gastrointestinal".ti,ab OR "gastro intestinal".ti,ab OR "intestinal".ti,ab OR "intestine".ti,ab OR "intestines".ti,ab OR "colon".ti,ab OR "rectum".ti,ab OR "anus".ti,ab OR "colonic".ti,ab OR "rectal".ti,ab OR "anal".ti,ab) AND ("stromal tumor".ti,ab OR "stromal tumors".ti,ab OR "stromal tumour".ti,ab OR "stromal tumours".ti,ab OR "stromal neoplasm".ti,ab OR "stromal neoplasms".ti,ab OR "stromal cancer".ti,ab))) AND ("neo adjuvant imatinib".ti,ab OR "neoadjuvant imatinib".ti,ab OR "neo adjuvant imatinib*".ti,ab OR "neoadjuvant imatinib*".ti,ab OR ((exp *"Imatinib"/ OR "Imatinib".ti,ab OR "Imatinib*".ti,ab OR "Gleevec".ti,ab OR "Glivec".ti,ab OR "multitargeted tyrosine kinase inhibitors".ti,ab OR "multitargeted tyrosine kinase inhibitor".ti,ab OR "multi targeted tyrosine kinase inhibitors".ti,ab OR "multi targeted tyrosine kinase inhibitor".ti,ab OR "tyrosine kinase inhibitors".ti,ab OR "tyrosine kinase inhibitor".ti,ab OR exp *"Protein Tyrosine Kinase inhibitor"/) AND (exp *"Neoadjuvant Therapy"/ OR "Neoadjuvant".ti,ab OR "Neoadjuvan*".ti,ab OR "Neo adjuvant".ti,ab OR "Neo adjuvan*".ti,ab OR exp *"Adjuvant Chemotherapy"/ OR "adjuvant".ti,ab))) AND (exp *"Computer assisted Tomography"/ OR "CT".ti,ab OR "computed tomography".ti,ab OR "computed tomography".ti,ab OR "computed tomogr*".ti,ab OR "computer tomogr*".ti,ab OR exp *"Positron Emission Tomography"/ OR "Positron-Emission Tomography".ti,ab OR "Positron-Emission Tomogr*".ti,ab OR "PET".ti,ab) AND (exp *"Patient Selection"/ OR "Patient Selection".ti,ab OR "Patients Selection".ti,ab OR "Subject Selection".ti,ab OR "Subjects Selection".ti,ab OR "Patient Recruitment".ti,ab OR "Patients Recruitment".ti,ab OR "Subject Recruitment".ti,ab OR "Subjects Recruitment".ti,ab OR "selection".ti,ab OR "select*".ti,ab OR "response evaluation".ti,ab OR "evaluate response".ti,ab OR "recruitment".ti,ab OR "recruit*".ti,ab OR exp *"Personalized Medicine"/ OR "personalized medicine".ti,ab OR "personalised medicine".ti,ab OR "personalized".ti,ab OR "personalised".ti,ab OR "prediction models".ti,ab OR "prediction model".ti,ab OR "prediction".ti,ab OR "predict*".ti,ab OR "monitoring".ti,ab OR "monitor".ti,ab OR "monitor*".ti,ab OR exp *"Genomics"/ OR "genomics".ti,ab OR "genomic".ti,ab OR "radiomics".ti,ab OR "genomic*".ti,ab OR exp *"Radiomics"/ OR "radiomic".ti,ab OR "radiomic*".ti,ab OR "machine learning".ti,ab OR exp *"Artificial Intelligence"/ OR exp *"Machine Learning"/ OR "Artificial Intelligence".ti,ab OR "Machine Learning".ti,ab OR "Deep Learning".ti,ab OR "prognost*".ti,ab OR "prognostics".ti,ab OR "prognostic factor".ti,ab OR "prognostic factors".ti,ab)) **OR** ((exp *"Gastrointestinal Stromal Tumor"/ OR "GIST tumor".ti OR "GIST tumors".ti OR "GIST tumour".ti OR "GIST tumours".ti OR "gastrointestinal tract stromal tumor".ti OR "gastrointestinal tract stromal tumors".ti OR "gastrointestinal stromal tumor".ti OR "gastrointestinal stromal tumors".ti OR "gastrointestinal stromal tumour".ti OR "gastrointestinal stromal tumours".ti OR "gastro intestinal stromal tumor".ti OR "gastro intestinal stromal tumors".ti OR "gastro intestinal stromal tumour".ti OR "gastro intestinal stromal tumours".ti OR (("gastrointestinal tract".ti OR "GI Tract".ti OR "GI".ti,ab OR "gastrointestinal".ti OR "gastro intestinal".ti OR "intestinal".ti OR "intestine".ti OR "intestines".ti OR "colon".ti OR "rectum".ti OR "anus".ti OR "colonic".ti OR "rectal".ti OR "anal".ti) AND ("stromal tumor".ti OR "stromal tumors".ti OR "stromal tumour".ti OR "stromal tumours".ti OR "stromal neoplasm".ti OR "stromal neoplasms".ti OR "stromal cancer".ti OR "stromal".ti))) AND (exp *"Patient Selection"/ OR "Patient Selection".ti,ab OR "Patients Selection".ti,ab OR "Subject Selection".ti,ab OR "Subjects Selection".ti,ab OR "Patient Recruitment".ti,ab OR "Patients Recruitment".ti,ab OR "Subject Recruitment".ti,ab OR "Subjects Recruitment".ti,ab OR "selection".ti,ab OR "select*".ti,ab OR "response evaluation".ti,ab OR "evaluate response".ti,ab OR "recruitment".ti,ab OR "recruit*".ti,ab OR exp *"Personalized Medicine"/ OR "personalized medicine".ti,ab OR "personalised medicine".ti,ab OR "personalized".ti,ab OR "personalised".ti,ab OR "prediction models".ti,ab OR "prediction model".ti,ab OR "prediction".ti,ab OR "predict*".ti,ab OR "monitoring".ti,ab OR "monitor".ti,ab OR "monitor*".ti,ab OR exp *"Genomics"/ OR "genomics".ti,ab OR "genomic".ti,ab OR exp *"Radiomics"/ OR "radiomics".ti,ab OR "genomic*".ti,ab OR "radiomic".ti,ab OR "radiomic*".ti,ab OR "machine learning".ti,ab OR exp *"Artificial Intelligence"/ OR "machine learning".ti,ab OR "Artificial Intelligence".ti,ab OR "Machine Learning".ti,ab OR "Deep Learning".ti,ab OR "prognost*".ti,ab OR "prognostics".ti,ab OR "prognostic factor".ti,ab OR "prognostic factors".ti,ab) AND (exp *"Computer Assisted Tomography"/ OR "CT".ti OR "computed tomography".ti OR "computed tomography".ti OR "computed tomogr*".ti OR "computer tomogr*".ti OR exp *"Positron-Emission Tomography"/ OR "Positron Emission Tomography".ti OR "Positron-Emission Tomogr*".ti OR "PET".ti))**)** NOT (("Case Report"/ OR "case report".ti) NOT (exp "Review"/ OR "review".ti OR "Clinical Study"/ OR exp "Clinical Trial"/ OR "trial".ti OR "RCT".ti))

Web of Science

(TS=(("Gastrointestinal Stromal Tumor" OR "GIST tumor" OR "GIST tumors" OR "GIST tumour" OR "GIST tumours" OR "gastrointestinal tract stromal tumor" OR "gastrointestinal tract stromal tumors" OR "gastrointestinal stromal tumor" OR "gastrointestinal stromal tumors" OR "gastrointestinal stromal tumour" OR "gastrointestinal stromal tumours" OR "gastro intestinal stromal tumor" OR "gastro intestinal stromal tumors" OR "gastro intestinal stromal tumour" OR "gastro intestinal stromal tumours" OR (("gastrointestinal tract" OR "GI Tract" OR "GI" OR "gastrointestinal" OR "gastro intestinal" OR "intestinal" OR "intestine" OR "intestines" OR "colon" OR "rectum" OR "anus" OR "colonic" OR "rectal" OR "anal") AND ("stromal tumor" OR "stromal tumors" OR "stromal tumour" OR "stromal tumours" OR "stromal neoplasm" OR "stromal neoplasms" OR "stromal cancer"))) AND ("neo adjuvant imatinib" OR "neoadjuvant imatinib" OR "neo adjuvant imatinib*" OR "neoadjuvant imatinib*" OR (("Imatinib" OR "Imatinib" OR "Imatinib*" OR "Gleevec" OR "Glivec" OR "multitargeted tyrosine kinase inhibitors" OR "multitargeted tyrosine kinase inhibitor" OR "multi targeted tyrosine kinase inhibitors" OR "multi targeted tyrosine kinase inhibitor" OR "tyrosine kinase inhibitors" OR "tyrosine kinase inhibitor" OR "Protein Tyrosine Kinase inhibitor") AND ("Neoadjuvant Therapy" OR "Neoadjuvant" OR "Neoadjuvan*" OR "Neo adjuvant" OR "Neo adjuvan*" OR "Adjuvant Chemotherapy" OR "adjuvant"))) AND ("Computer assisted Tomography" OR "CT" OR "computed tomography" OR "computer tomography" OR "computed tomogr*" OR "computer tomogr*" OR "Positron Emission Tomography" OR "Positron-Emission Tomography" OR "Positron-Emission Tomogr*" OR "PET") AND ("Patient Selection" OR "Patient Selection" OR "Patients Selection" OR "Subject Selection" OR "Subjects Selection" OR "Patient Recruitment" OR "Patients Recruitment" OR "Subject Recruitment" OR "Subjects Recruitment" OR "selection" OR "select*" OR ″response evaluation″ OR ″evaluate response″ OR "recruitment" OR "recruit*" OR "Personalized Medicine" OR "personalized medicine" OR "personalised medicine" OR "personalized" OR "personalised" OR "prediction models" OR "prediction model" OR ″predict*″ OR "prediction" OR "monitoring" OR "monitor" OR "monitor*" OR "Genomics" OR "genomics" OR "genomic" OR "radiomics" OR "genomic*" OR "Radiomics" OR "radiomic" OR "radiomic*" OR "machine learning" OR "Artificial Intelligence" OR "Machine Learning" OR "Artificial Intelligence" OR "Machine Learning" OR "Deep Learning" OR ″prognost*″ OR "prognostics" OR "prognostic factor" OR "prognostic factors")) **OR** (ti=("Gastrointestinal Stromal Tumor" OR "GIST tumor" OR "GIST tumors" OR "GIST tumour" OR "GIST tumours" OR "gastrointestinal tract stromal tumor" OR "gastrointestinal tract stromal tumors" OR "gastrointestinal stromal tumor" OR "gastrointestinal stromal tumors" OR "gastrointestinal stromal tumour" OR "gastrointestinal stromal tumours" OR "gastro intestinal stromal tumor" OR "gastro intestinal stromal tumors" OR "gastro intestinal stromal tumour" OR "gastro intestinal stromal tumours" OR (("gastrointestinal tract" OR "GI Tract" OR "GI" OR "gastrointestinal" OR "gastro intestinal" OR "intestinal" OR "intestine" OR "intestines" OR "colon" OR "rectum" OR "anus" OR "colonic" OR "rectal" OR "anal") AND ("stromal tumor" OR "stromal tumors" OR "stromal tumour" OR "stromal tumours" OR "stromal neoplasm" OR "stromal neoplasms" OR "stromal cancer" OR "stromal"))) AND ts=("Patient Selection" OR "Patient Selection" OR "Patients Selection" OR "Subject Selection" OR "Subjects Selection" OR "Patient Recruitment" OR "Patients Recruitment" OR "Subject Recruitment" OR "Subjects Recruitment" OR "selection" OR "select*" OR ″response evaluation″ OR ″evaluate response″ OR "recruitment" OR "recruit*" OR "Personalized Medicine" OR "personalized medicine" OR "personalised medicine" OR "personalized" OR "personalised" OR "prediction models" OR "prediction model" OR ″predict*″ OR "prediction" OR "monitoring" OR "monitor" OR "monitor*" OR "Genomics" OR "genomics" OR "genomic" OR "Radiomics" OR "radiomics" OR "genomic*" OR "radiomic" OR "radiomic*" OR "machine learning" OR "Artificial Intelligence" OR "machine learning" OR "Artificial Intelligence" OR "Machine Learning" OR "Deep Learning" OR ″prognost*″ OR "prognostics" OR "prognostic factor" OR "prognostic factors") AND ti=("Computer Assisted Tomography" OR "CT" OR "computed tomography" OR "computed tomography" OR "computed tomogr*" OR "computer tomogr*" OR "Positron-Emission Tomography" OR "Positron Emission Tomography" OR "Positron-Emission Tomogr*" OR "PET"))**)** NOT ti=(("Case Report" OR "case report") NOT ("Review" OR "review" OR "Clinical Study" OR "Clinical Trial" OR "trial" OR "RCT"))

Cochrane

((("Gastrointestinal Stromal Tumor" OR "GIST tumor" OR "GIST tumors" OR "GIST tumour" OR "GIST tumours" OR "gastrointestinal tract stromal tumor" OR "gastrointestinal tract stromal tumors" OR "gastrointestinal stromal tumor" OR "gastrointestinal stromal tumors" OR "gastrointestinal stromal tumour" OR "gastrointestinal stromal tumours" OR "gastro intestinal stromal tumor" OR "gastro intestinal stromal tumors" OR "gastro intestinal stromal tumour" OR "gastro intestinal stromal tumours" OR (("gastrointestinal tract" OR "GI Tract" OR "GI" OR "gastrointestinal" OR "gastro intestinal" OR "intestinal" OR "intestine" OR "intestines" OR "colon" OR "rectum" OR "anus" OR "colonic" OR "rectal" OR "anal") AND ("stromal tumor" OR "stromal tumors" OR "stromal tumour" OR "stromal tumours" OR "stromal neoplasm" OR "stromal neoplasms" OR "stromal cancer"))) AND ("neo adjuvant imatinib" OR "neoadjuvant imatinib" OR "neo adjuvant imatinib*" OR "neoadjuvant imatinib*" OR (("Imatinib" OR "Imatinib" OR "Imatinib*" OR "Gleevec" OR "Glivec" OR "multitargeted tyrosine kinase inhibitors" OR "multitargeted tyrosine kinase inhibitor" OR "multi targeted tyrosine kinase inhibitors" OR "multi targeted tyrosine kinase inhibitor" OR "tyrosine kinase inhibitors" OR "tyrosine kinase inhibitor" OR "Protein Tyrosine Kinase inhibitor") AND ("Neoadjuvant Therapy" OR "Neoadjuvant" OR "Neoadjuvan*" OR "Neo adjuvant" OR "Neo adjuvan*" OR "Adjuvant Chemotherapy" OR "adjuvant"))) AND ("Computer assisted Tomography" OR "CT" OR "computed tomography" OR "computed tomography" OR "computed tomogr*" OR "computer tomogr*" OR "Positron Emission Tomography" OR "Positron Emission Tomography" OR "Positron Emission Tomogr*" OR "PET") AND ("Patient Selection" OR "Patient Selection" OR "Patients Selection" OR "Subject Selection" OR "Subjects Selection" OR "Patient Recruitment" OR "Patients Recruitment" OR "Subject Recruitment" OR "Subjects Recruitment" OR "selection" OR "select*" OR ″response evaluation″ OR ″evaluate response″ OR "recruitment" OR "recruit*" OR "Personalized Medicine" OR "personalized medicine" OR "personalised medicine" OR "personalized" OR "personalised" OR "prediction models" OR "prediction model" OR ″predict*″ OR "prediction" OR "monitoring" OR "monitor" OR "monitor*" OR "Genomics" OR "genomics" OR "genomic" OR "radiomics" OR "genomic*" OR "Radiomics" OR "radiomic" OR "radiomic*" OR "machine learning" OR "Artificial Intelligence" OR "Machine Learning" OR "Artificial Intelligence" OR "Machine Learning" OR "Deep Learning" OR ″prognost*″ OR "prognostics" OR "prognostic factor" OR "prognostic factors")):ti,ab,kw **OR** (ti=("Gastrointestinal Stromal Tumor" OR "GIST tumor" OR "GIST tumors" OR "GIST tumour" OR "GIST tumours" OR "gastrointestinal tract stromal tumor" OR "gastrointestinal tract stromal tumors" OR "gastrointestinal stromal tumor" OR "gastrointestinal stromal tumors" OR "gastrointestinal stromal tumour" OR "gastrointestinal stromal tumours" OR "gastro intestinal stromal tumor" OR "gastro intestinal stromal tumors" OR "gastro intestinal stromal tumour" OR "gastro intestinal stromal tumours" OR (("gastrointestinal tract" OR "GI Tract" OR "GI" OR "gastrointestinal" OR "gastro intestinal" OR "intestinal" OR "intestine" OR "intestines" OR "colon" OR "rectum" OR "anus" OR "colonic" OR "rectal" OR "anal") AND ("stromal tumor" OR "stromal tumors" OR "stromal tumour" OR "stromal tumours" OR "stromal neoplasm" OR "stromal neoplasms" OR "stromal cancer" OR "stromal"))):ti AND ts=("Patient Selection" OR "Patient Selection" OR "Patients Selection" OR "Subject Selection" OR "Subjects Selection" OR "Patient Recruitment" OR "Patients Recruitment" OR "Subject Recruitment" OR "Subjects Recruitment" OR "selection" OR "select*" OR ″response evaluation″ OR ″evaluate response″ OR "recruitment" OR "recruit*" OR "Personalized Medicine" OR "personalized medicine" OR "personalised medicine" OR "personalized" OR "personalised" OR ″predict*″ OR "prediction models" OR "prediction model" OR "prediction" OR "monitoring" OR "monitor" OR "monitor*" OR "Genomics" OR "genomics" OR "genomic" OR "Radiomics" OR "radiomics" OR "genomic*" OR "radiomic" OR "radiomic*" OR "machine learning" OR "Artificial Intelligence" OR "machine learning" OR "Artificial Intelligence" OR "Machine Learning" OR "Deep Learning" OR ″prognost*″ OR "prognostics" OR "prognostic factor" OR "prognostic factors"):ti,ab,kw AND ti=("Computer Assisted Tomography" OR "CT" OR "computed tomography" OR "computed tomography" OR "computed tomogr*" OR "computer tomogr*" OR "Positron Emission Tomography" OR "Positron Emission Tomography" OR "Positron Emission Tomogr*" OR "PET"):ti)**)**

Supplementary materials: Quality assessment

Modified radiomics Quality Score (RQS_m):

Q1: Image protocol quality – well-documented imaging protocols (for example, contrast, slice thickness, energy, etc).

- Protocols well documented (+1)
- None (+0)

Q2: Multiple segmentations – possible actions are: segmentation by different physicians/algorithms/software, perturbing segmentation by (random) noise, segmentation at different breathing cycles. Analyse robustness to segmentation variabilities.

- Yes (+1)
- No (+0)

Q3: Phantom study on all scanners – detect inter-scanner differences and vendor-dependent features. Analyse feature robustness to these sources of variability.

- Yes (+1)
- No (+0)

Q4: Feature reduction or adjustment for multiple testing – decreases the risk of overfitting. Overfitting is inevitable if the number of features exceeds the number of samples. Consider feature robustness when selecting features.

- Either measure is implemented (+3)
- Neither measure is implemented (-3)

Q5: Detect and discuss biological correlates – demonstration of phenotypic differences (possibly associated with underlying gene-protein expression patterns) deepens understanding of radiomics and biology.

- Yes (+1)
- No (+0)

Q6: Cut-off analyses – determine risk groups by either the median, previously published cut-off or report a continuous risk variable. Reduces the risk of reporting overly optimistic results.

- Yes (+1)
- No (+0)

Q7: Discrimination statistics – report discrimination statistics (for example, C-statistics, ROC curve, AUC) and their statistical significance (for example, p-values, confidence intervals). One can also apply resampling method (for example, bootstrapping, cross-validation).

- A discrimination statistic and its statistical significance are reported (+1)
- A resampling method technique is also applied (+1)
- None (+0)

Q8: Calibration statistics - report calibration statistics (for example, Calibration-in-the-large/slope, calibration plots) and their statistical significance (for example, P-values, confidence

intervals). One can also apply resampling method (for example, bootstrapping, cross-validation).

- A calibration statistic and its statistical significance are reported (+1)
- A resampling method technique is applied (+1)
- None (+0)

Q9: Validation – the validation is performed without retraining and without adaptation of the cut-off value, provides crucial information with regard to credible clinical performance.

- No validation (-5)
- Validation is based on a dataset from the same institute (+1)
- Validation is based on a dataset from another institute (+2)
- Validation is based on two datasets from two distinct institutes (+3)
- The study validates a previously published signature (+4)
- Validation is based on three or more datasets from distinct institutes (+5)

Q10: Comparison to 'gold standard' - assess the extent to which the model agrees with/is superior to the current 'gold standard' method (for example, TNM-staging for survival prediction). This comparison shows the added value of radiomics.

- Yes (+2)
- No (+0)

Q11: Potential clinical utility - report on the current and potential application of the model in a clinical setting (for example, decision curve analysis).

- Yes (+2)
- No (+0)

Q12: Cost-effectiveness analysis - report on the cost-effectiveness of the clinical application (for example, QALYs generated).

- Yes (+1)
- No (+0)

Q13: Open science and data - make code and data publicly available. Open science facilitates knowledge transfer and reproducibility of the study

- Scans are open source (+1)
- Region of interest segmentations are open source (+2)
- Code is open sourced (+3)
- Radiomics features are calculated on a set of representative ROIs
and the calculated features and representative ROIs are open source. (+4)

Modified Radiomics Quality Score (RQS) for non-radiomic studies ($RQS_{m,nonrad}$)

Q1: Image protocol quality – well-documented imaging protocols (for example, contrast, slice thickness, energy, etc).

- Protocols well documented (+1)
- None (+0)

Q2: Interobserver or inter-scanner variability – Have these variabilities been taken into account? (two observers in consensus reading or the use of a standardised acquisition protocol)

- Yes (+1)
- No (+0)

Q3: Detect and discuss biological correlates – demonstration of phenotypic differences (possibly associated with underlying gene-protein expression patterns) deepens understanding of predictors and biology.

- Yes (+1)
- No (+0)

Q4: Cut-off analyses – determining risk groups by either the median, previously published cut-off or report a continuous risk variable. Reduces the risk of reporting overly optimistic results.

- Yes (+1)
- No (+0)

Q5: Discrimination statistics – report discrimination statistics (for example, C-statistics, ROC curve, AUC) and their statistical significance (for example, p-values, confidence intervals). One can also apply resampling method (for example, bootstrapping, cross-validation).

- Yes (+1)
- No (+0)

Q6: Validation – Outcomes have been validated on an unseen dataset, providing crucial information with regard to credible clinical performance.

- Cross-validation (+1)
- Test set (+2)
- None (+0)

Q7: Comparison to 'gold standard' - assess the extent to which the prediction model/predictors agrees with/is superior to the current 'gold standard' method (for example, TNM-staging for survival prediction).

- Yes (+1)
- No (+0)

Q8: Potential clinical utility - report on the current and potential application of the model in a clinical setting. Decision-curve analysis is in this case not essential, but one should mention how it could affect routine-clinical practice (for example, therapeutic regime switches)

- Yes (+1)

- No (+0)

Q9: Open science and data - make code and data publicly available. Open science facilitates knowledge transfer and reproducibility of the study.
- Scans/ROI's/features/code open source (+1)
- None (+0)

Supplementary materials: Study characteristics

Table 1 | Predicting mutational status

Author	Year	Patient group	Features	Results on efficacy	Quality score
[18F]FDG) PET/CT					
Fuster et al.	2011	Localized and advanced refractory (n = 15)	SUVmax	Patients with KIT-WT showed a significantly lower SUVmax at baseline compared to non-WT KIT using univariate analysis (p < 0.05).	Low quality 3/10
Kwon et al.	2019	Localized (n = 12)	Retention index	The retention index was significantly higher in KIT mutated tumors compared to non-KIT tumors, using univariate analysis (p = 0.046).	Low quality 3/10
CE-CT imaging					
Xu et al.	2018	Localized and advanced (n = 86)	StdDeviation<br>+ tumour location and CD34 stain level	Radiomics model predicting the presence of KIT exon 11 mutation with AUCs of 0.904-0.962, which was higher compared to subjective visual assessment.	Low quality 11/26
Yin et al.	2019	Localized and advanced small intestine (n = 35)	Tumour enhancement ratio	Using a 1.60 cut-off point for enhancement ratio resulted into an AUC, sensitivity and specificity of 76.0%, 86.7% and 98.5% for differentiating exon 9 from exon 11 mutants.	High quality 5/10
Cannella et al.	2021	Localized and advanced (n = 88)	Enhancement degree	Hyperenhancement was significantly more frequent in PDGFRα-mutated/wild-type GISTS compared to KIT mutations using univariate analysis (p = 0.004).	Low quality 3/10
Liu et al.	2021	Localized and advanced (n = 327)	40 features (radiomic + clinical + subjective CT features)<br>24 features (radiomic + clinical + subjective CT features)	Model for prediction of KIT exon 11 mutation with an AUC of 0.811, while the model for prediction of KIT exon 11 mutation with deletion achieved an AUC of 0.849.	High quality 13/26
Liu et al.	2022	Localized and advanced (n = 106)	Nine features (first-order, shape and texture)	Preoperative nomogram with AUC, sensitivity and specificity of 0.715, 80,0% and 72.7% in distinguishing KIT exon 11 mutations from non-KIT exon mutations.	High quality 17/26
Palatresi et al.	2022	Localized (n = 54)	Three features (first-order and texture)	All features considered predictors for determining KIT exon 11 or 9/PDGFR-α exon 12 or 18 mutations. (p = 0.026, p = 0.048, p = 0.026 and p = 0.047).	Low quality 4/10
Starmans et al.	2022	Localized and advanced (n = 98)	42 features (first-order and texture)<br>+ age, sex and tumour location	Radiomics model predicting KIT and KIT exon 11 presence with 0.510 and 0.570 AUC, 0.960 and 70.0% sensitivity and 3.0% and 36.0% specificity, respectively.	High quality 15/26

Table 1. Summarized overview of the included articles on the prediction of mutational status, including study characteristics, quality score, evaluated radiological features and their conclusions on efficacy (SUV = standardized uptake value, WT = wild type, AUC = area under the curve and PDGFR = platelet derived growth factor receptor)

Table 2.1 | **Predicting mitotic index**

Author	Year	Patient group	Features	Results on efficacy	Quality score
[18F]FDG) PET/CT					
Kamiyama et al.	2005	Localized gastric (n = 10)	SUVmax	SUVmax was significantly higher in high mitotic GISTs, using univariate analysis ($p = 0.05$).	Low quality 4/10
Park et al.	2011	Localized gastric (n = 26)	SUVmax	SUVmax was significantly higher in high mitotic GISTs, using univariate analysis ($p = 0.05$).	Low quality 3/10
Yoshikawa et al.	2013	Localized (n = 10)	SUVmax	SUVmax was significantly higher in high mitotic GISTs, using univariate analysis ($p = 0.029$).	Low quality 2/10
Tokumoto et al.	2014	Localized gastric (n = 30)	SUVmax	No significant correlation with high SUV and high mitotic count, using univariate analysis.	Low quality 4/10
Miyake et al.	2016	Localized (n = 46)	Uptake patterns	The mitotic index for GISTs with ring-shaped uptake was significantly higher, using univariate analysis ($p = 0.017$).	Low quality 3/10
Kwon et al.	2019	Localized (n = 32)	SUV after one and two hour(s) and retention index	SUV1, SUV2 and retention index were significantly higher in high mitotic GISTs ($p = 0.041$, $p = 0.041$, $p = 0.031$).	Low quality 3/10
CE-CT imaging					
Kim et al.	2004	Localized and advanced gastric (n = 81)	Tumor size	Tumor size was a significant predictor of high mitotic rate (OR, 2.57; CI; 1.42-4.67).	Low quality 4/10
Kim et al.	2005	Localized gastric (n = 39)	Density (areas of low attenuation)	Small GISTs (≤ 5 cm) with areas of low attenuation did not have a significantly higher mitotic rate, using univariate analysis.	Low quality 4/10
Ulusan et al.	2008	Localized and advanced (n = 30)	Tumor size, location, enhancement pattern, metastasis and necrosis	Heterogeneous enhancement, tumor size, stomach location, presence of necrosis and metastasis were associated with a high mitotic index (all p-values < 0.05).	Low quality 3/10
Al-balas et al.	2012	Localized and advanced (n = 26)	Enhancement pattern and necrosis	Heterogeneous enhancement and presence of necrosis were not predictive for high mitotic count.	Low quality 1/10
Pelandré et al.	2013	Localized and advanced gastric (n = 21)	Shape and infiltration	Irregular shape and presence of mesenteric fat infiltration correlated with high mitotic index, using univariate analysis ($p = 0.027$).	Low quality 4/10
Pinaikul et al.	2014	Localized and advanced (n = 50)	Necrosis and peritoneal seeding	Presence of necrosis and peritoneal seeding were significant predictors for high mitotic rate ($p < 0.05$).	Low quality 3/10
Iannicelli et al.	2017	Localized and advanced (n = 44)	Tumor shape	Irregular tumor outline was associated with high mitotic index using univariate analysis ($p = 0.016$).	Low quality 4/10
Chen et al.	2019	Localized gastric (n = 50)	Tumor shape and growth pattern	Irregular tumor shape and exophytic/mixed growth pattern were associated with high mitotic count using univariate analysis ($p = 0.009$).	Low quality 4/10
Wang et al.	2019	Localized (n = 333)	Fourteen features (first-order, shape and texture)	Radiomic model discriminating high from low mitotic GISTs with 0.769 AUC, 52.4% sensitivity, 81.0% specificity and 75.0% accuracy.	Low quality 12/26
Wei et al.	2020	Localized (n = 101)	Tumor shape + Ki-67 index	Irregular shape and Ki-67 index are both predictive factors for high mitotic value with an accuracy of 0.878.	High quality 5/10
Chen et al.	2020	Localized 2-5 cm gastric (n = 60)	Enhancement pattern ratio (PVP/delayed)	Using a 0.99 cut-off point for the ratio between HU's on portal and delayed phase , an AUC of 0.722 was achieved.	High quality 5/10
Mazzei et al.	2020	Localized and advanced (n = 42)	Density (areas of low attenuation)	With a cut-off of 20% for hypodensity, high mitotic count was predicted with 91.6% accuracy.	High quality 5/10
Starmans et al.	2022	Localized and advanced (n = 90)	42 features (first-order and texture) + age, sex and tumor location	Radiomic model predicting mitotic index with 0.540 AUC, 27.0% sensitivity and 75.0% specificity.	High quality 15/26

Table 2.1. Summarized overview of the included articles on the prediction of mitotic index (proliferative activity), including study characteristics, quality score, evaluated radiological features and their conclusions on efficacy (SUV = standardized uptake value, OR = odds ratio, AUC = area under the curve and GIST = gastrointestinal stromal tumors)

Table 2.2| Predicting Ki-67 proliferation index

Author	Year	Patient group	Features	Results on efficacy	Quality score
[18F]FDG) PET/CT					
Kamiyama et al.	2005	Localized gastric (n = 10)	SUVmax	Significant correlation between Ki-67 index and SUVmax (r =0.832, p = 0.002).	Low quality 4/10
Park et al.	2011	Localized gastric (n = 26)	SUVmax	SUVmax was correlated with Ki-67 index, using univariate analysis (r = 0.8854, p = 0.000).	Low quality 3/10
Yoshikawa et al.	2013	Localized (n = 10)	SUVmax	Significant correlation between Ki-67 index and SUVmax (r = 0.680, p = 0.028) using univariate analysis.	Low quality 2/10
Kurata et al	2018	Localized (n = 64)	Fractal dimension and SUVmax	Fractal dimension and SUVmax predictive for high Ki-67 index with sensitivities of both 66.7% and specificities of 69.8% and 92.3%, respectively.	Low quality 3/10
CE-CT imaging					
Li et al.	2018	Localized and advanced (n = 151)	Tumor size and ulceration	Tumor size and ulceration were both significantly different between high and low Ki67 index groups (p = 0.043, p = 0.011). Using a cut-off point of 5.75 cm for Tumor size, an AUC of 0.726 was achieved.	Low quality 4/10
Zhang et al.	2020	Localized (n = 339)	Six features (first-order and texture) + tumor size	The radiomic nomogram achieved an AUC, sensitivity, specificity and accuracy of 0.784, 58.8%, 77.6% and 73.3% in predicting high Ki-67 index.	High quality 18/26
Zhao et al.	2021	Localized (n = 344)	Twenty-one features (first-order, shape and texture)	The AUC value of the radiomic model was 0.784 for predicting high Ki-67 index.	High quality 13/26
Zhu et al.	2021	Localized and advanced small intestine (n = 123)	Tumor size, metastasis and ulceration	Using tumor size and presence of metastasis and ulceration resulted in an AUC, sensitivity, specificity and accuracy of 0.785, 63.3%, 76.3% and 73.2%.	High quality 5/10
Chen et al.	2021	Localized gastric (n = 167)	Shape and necrosis volume ratio	Irregular/lobulated shape and high necrosis volume ratio indicated high-level Ki-67 index (p < 0.001 and p = 0.024).	Low quality 4/10
Yang et al.	2021	Localized and advanced (n = 198)	Necrosis, cystic generation and enhancement degree	Presence of cystic generation, necrosis and hyperenhancement of overlying mucosa were independent predictive factors for high Ki-67 index (p = 0.049, p < 0.001 and p , 0.001).	High quality 5/10
Feng et al.	2022	Localized (n = 382)	Nineteen features + tumor size, growth pattern and ulceration	Nomogram results into AUC of 0.772, which was significantly higher than a model using CT subjective findings (p=0.0098).	High quality 15/26

Table 2.2. Summarized overview of the included articles on the prediction of Ki-67 proliferation index (proliferative activity), including study characteristics, quality score, evaluated radiological features and their conclusions on efficacy (SUV = standardized uptake value and AUC = area under the curve)

Table 3.1 | **Predicting modified National Institutes of Health (NIH) criteria**

Author	Year	Patient group	Features	Results on efficacy	Quality score
[^{18}F]FDG) PET/CT					
Miyake et al.	2016	Localized (n = 46)	Uptake patterns	Ring-shaped and intense uptake were significantly associated with Joensuu high risk GISTs using univariate analysis (p = 0.015, p = 0.008).	Low quality 3/10
Hwang et al.	2021	Localized (n = 62)	MTV and TLG	In time-dependent ROC analysis MTV and TLG combined showed better predictive accuracy than Joensuu criteria (AUC's 0.76 vs. 0.86 and 0.87).	High quality 5/10
CE-CT imaging					
Zhou et al.	2016	Localized (n = 129)	Tumor size, growth pattern and feeding vessels	Tumor size > 10 cm, mixed growth pattern or presence of feeding vessels indicated higher risk GISTs with an AUC of 0.806.	High quality 5/10
Li et al.	2018	Localized and advanced (n = 151)	Calcification and enhancement pattern	Presence of calcification and enhancement degree showed no significant difference between risk grades.	Low quality 4/10
Chen et al	2019	Localized (n = 222)	Ten features (texture) + tumor diameter, hemorrhage, growth pattern and enlarged feeding vessels	Radiomic nomogram used to predict high-risk GISTs with an AUC of 0.867.	High quality 15/26
Chen et al.	2019	Localized gastric (n = 50)	Tumor size and growth pattern	Tumor size ≥ 5 cm and exophytic/mixed growth are indicators of higher grade tumors (p = 0.002 and p = 0.011).	Low quality 4/10
Ren et al.	2019	Localized (n = 168)	Four features (shape and texture) + tumor size and location	Radiomic model for high risk prediction, resulting in 0.959 AUC, 90.5% sensitivity, 93.3% specificity and 90.2% accuracy.	High quality 13/26
Wang et al	2019	Localized (n = 333)	Two features (max diameter and intensity values range) + tumor location	Radiomic model discriminating high from low risk grade with 0.920 AUC, 76,3% sensitivity, 88,7%specificity and 84.0% accuracy.	Low quality 12/26
Wei et al.	2020	Localized and advanced (n = 101)	Angle between the long and short tumor diameter.	Using a 90.5° cut-off for the angle, 0.852 AUC, 0.824 sensitivity, 0.871 specificity and 0.825 was achieved.	High quality 5/10
Xu et al.	2020	Localized gastric (n = 150)	Tumor size, growth pattern, shape, enhancement pattern and enlarged vessels.	Multi-class radiomic model with AUC of 0.912 and 0,972 for predicting very low and high risk GISTs, respectively.	Low quality 11/26
Zhang et al.	2020	Localized (n = 366)	Forty-eight (first-order, shape and texture).	Radiomic signature with an AUC of 0.935 for discriminating low from high risk GIST.	Low quality 10/26
Ren et al.	2020	Localized (n = 440)	Density (mean value) + Tumor size and cystoid variation	Nomogram discriminating low from high risk GISTs with 0.933 AUC, 90.6% sensitivity, 75.7% specificity and 88.6% accuracy.	High quality 16/26
Zhang et al.	2020	Localized (n =370)	Thirteen features (first-order, shape and texture)	The radiomic model achieved an AUC of 0.899 when predicting high risk GISTs.	High quality 13/26
Cannella et al.	2021	Localized and advanced (n = 88)	Tumor size and enlarged feeding vessels.	Tumor size ≥ 5 cm and presence of enlarged feeding vessels were predictors for high-risk GISTs (p = 0.009 and p = 0.048).	Low quality 3/10
Li et al.	2021	Localized gastric (n = 206)	Tumor size, feeding vessels, location and infiltration	Nomogram using tumor size > 5 cm, cardiac location and presence of feeding vessels and infiltration showed an AUC, sensitivity and specificity of 0.946, 0.896 and 0.915.	High quality 6/10
Wang et al.	2021	Localized (n = 324)	Ten features (shape and texture)	Random forest model with 84.0% accuracy, 93.0% sensitivity, 76,0% specificity and 0.90 AUC.	High quality 14/26
Peng et al.	2021	Localized and advanced gastric (n = 147)	Overlying gastric mucosa	Incomplete overlying enhancing gastric mucosa was predictive for high-risk grade with an AUC, accuracy, specificity and sensitivity of 0.835, 82.3%, 77.0% and 90.0%.	High quality 6/10
Zhu et al.	2021	Localized and advanced small intestine (n = 123)	Tumor size and necrosis	Tumor size combined with the presence of necrosis achieved an AUC, sensitivity, specificity and accuracy of 0.965, 91.5%, 96.9% and 94,3%.	High quality 5/10
Chen et al.	2021	Localized (n = 381)	Fourteen features (shape and texture)	Three-class radiomic model with AUC's of 0.880, 0.780 and 0.830 for predicting low, intermediate and high risk, respectively.	High quality 13/26
Chu et al.	2021	Localized (n = 292)	Ten features (shape and texture)	AUC of 0.791 with 0.842 sensitivity, 0.693 specificity and 0.759 accuracy in predicting high risk GISTs, outperforming the clinical model.	High quality 13/26
Kang et al.	2021	Localized (n = 733)	Deep learning	Three-class deep learning model achieved AUC of 0.87, 0.64 and 0.85 for low, intermediate and high risk GIST prediction, outperforming a concurrent radiomic	Low quality 12/26
Shao et al.	2021	Localized gastric (n = 231)	Fifteen features (shape and texture) + LD, SD and shape	Discriminating risk rating using radiomic model to achieve 0.897 AUC , 88.4% accuracy, 88.6% sensitivity and 88.2% specificity.	Low quality 8/26
Tang et al.	2022	Localized (n = 326)	Tumor size, necrosis, ulceration and portal venous phase minus arterial phase (PVPMAP).	Models to predict high risk GISTs for gastric and small bowel achieved AUC of 0.958 and 0.921, respectively.	High quality 5/10

Table 3.1 Summarized overview of the included articles on risk stratification using the modified National Institutes of Health (NIH) criteria, including study characteristics, quality score, evaluated radiological features and their conclusions on efficacy (SUV = standardized uptake value and, MTV = metabolic tumor volume, TLG = total lesion glycolysis, AUC = area under the curve and GIST = gastrointestinal stromal tumor)

Table 3.2 | **Predicting National Institutes of Health (NIH) consensus criteria**

Author	Year	Patient group	Features	Results on efficacy	Quality score
**[^{18}F]FDG) PET/CT**					
Otomi et al.	2010	Localized (n = 11)	SUVmax	The baseline SUVmax was significantly higher in the high risk group compared to low/intermediate group (p < 0.001), using univariate analysis.	Low quality 2/10
Park et al.	2011	Localized gastric (n = 26)	SUVmax	With a 3.94 cut-off, sensitivity and specificity were 85.7% and 94.7%, using univariate analysis.	Low quality 3/10
Yoshikawa et al.	2013	Localized (n = 10)	SUVmax	Significant difference between low/intermediate and high risk groups in SUVmax (p < 0.05), using univariate analysis.	Low quality 2/10
Tokumoto et al.	2014	Localized gastric (n = 30)	SUVmax	With a threshold > 3.0 g/ml, the sensitivity and specificity were 85,7% and 62.5% for predicting high-risk groups.	Low quality 4/10
Cho et al.	2015	Localized (n = 40)	SUVmax	Using a 4.99 cut-off , 0.875 AUC, 89.5%sensitivity, 76.2% specificity and 82.5% accuracy was achieved.	Low quality 4/10
Kurata et al	2018	Localized (n = 64)	SUVmax	SUVmax is significantly higher in high-risk groups (p < 0.01).	Low quality 3/10
Kwon et al.	2019	Localized (n = 32)	SUVmax one hour post-injection	A cut-off of 5.2 g/ml demonstrated a sensitivity of 90.0% and specificity of 89.0% for predicting high risk GISTs.	Low quality 3/10
Albano et al.	2020	Localized (n = 35)	SUV corrected for body weight, lean body mass, surface area, MTV and TLG.	Metabolic parameters were significantly correlated with tumor risk group using, univariate analysis (p = 0.016, p = 0.013, p = 0.017 and p = 0.009).	High quality 6/10
**CE-CT imaging**					
Wang et al.	2017	Localized (n = 100)	Ten semantic CT findings	Three-class support vector machine model to classify high, intermediate and low risk grades with an accuracy of 70.0%.	High quality 5/10
Kurata et al	2018	Localized (n = 64)	Fractal dimension	Fractal dimension is significantly higher in high-risk groups (p < 0.01), when combined with SUVmax it lead to sensitivities, specificities and accuracies above 71.0%.	Low quality 3/10

Table 3.2. Summarized overview of the included articles on risk stratification using the National Institutes of Health (NIH) consensus criteria, including study characteristics, quality score, evaluated radiological features and their conclusions on efficacy (SUV = standardized uptake value, MTV = metabolic tumor volume, TLG = total lesion glycolysis and AUC = are under the curve).

Table 3.3 | **Predicting American Forces Institute of Pathology (AFIP) criteria**

Author	Year	Patient group	Features	Results on efficacy	
**CE-CT imaging**					
Yang et al.	2007	Localized and advanced (n = 39)	Tumor size	Larger tumor size (≥ 5 cm) was significant for high-risk GISTs (p < 0.05).	Low quality 2/10
Iannicelli et al.	2017	Localized and advanced (n = 44)	Tumor shape, enhancement patterns, feeding vessels, necrosis and adjacent organ invasion	Heterogeneous enhancement, ill-defined borders, presence of necrosis, feeding vessels and organ invasion are associated with classes of risk in univariate analysis (p = 0.002, p = 0.006, p = 0.006, p = 0.006 and p = 0.011).	Low quality 4/10
Liu et al.	2018	Localized (n = 78)	Ten features (first-order statistics)	All features showed significant diagnostic performance with AUC's between 0.636 and 0.811, where max frequency performed best.	High quality 5/10
Choi et al	2019	Localized (n = 144)	Mean of positive pixels	With a cut-off point of 49.3, high grade was predicted with 0.782 AUC, 0.887 sensitivity and 0.750 specificity, similar to visual inspection by radiologists.	High quality 6/10
Grazzini et al.	2021	Localized and advanced (n = 54)	Necrosis and feeding vessels	Presence of both factors predicted high pathological risk with 90.5% sensitivity, 88.6% specificity and 89.3% accuracy.	Low quality 3/10
Palatresi et al.	2022	Localized (n = 54)	Five features (shape and texture)	All features considered predictors for differentiating high from low risk GISTs (p = 0.043, p = 0.034, p = 0.027, p = 0.043 and p = 0.027).	Low quality 4/10

Table 3.3. Summarized overview of the included articles on risk stratification using the American Forces Institute of Pathology (AFIP) criteria, including study characteristics, quality score, evaluated radiological features and their conclusions on efficacy (AUC= are under the curve and GIST = gastrointestinal stromal tumor).

Table 3.4 | **Predicting other risk stratifications**

Author	Year	Patient group	Features	Results on efficacy	
**CE-CT imaging**					
Tateishi et al.	2003	Localized and advanced (n = 69)	Tumor size, shape, adjacent organ invasion, enhancement patterns, metastasis and peritoneal seeding.	Larger tumors (> 11.1 cm), irregular outline, presence of invasion, heterogeneous enhancement, hepatic metastasis, and peritoneal dissemination were all significant CT findings for high-grade GISTs.	Low quality 3/10
Verde et al.	2017	Localized small bowel (n = 22)	Enhancement pattern and necrosis	Heterogeneous enhancement and presence of necrosis were significantly associated with TNM risk-grade using univariate analysis (p = 0.001 and p < 0.001).	Low quality 3/10

Table 3.4. Summarized overview of the included articles on less common risk stratification criteria, including study characteristics, quality score, evaluated radiological features and their conclusions on efficacy (TNM = tumor, node, metastases and GIST = gastrointestinal stromal tumor).

Table 4 | **Predicting radiological response**

Author	Year	Patient group	Features	Results on efficacy	Quality score
**CE-CT imaging**					
Ekert et al.	2019	Advanced (n = 25)	Four features (texture)	Combining features to predict disease progression with an AUC of 0.827.	Low quality 5/10

Table 4 Summarized overview of the included articles predicting radiological response, including study characteristics, quality score, evaluated radiological features and their conclusions on efficacy (AUC = area under the curve)

Table 5 | **Predicting prognosis**

Author	Year	Patient group	Features	Results on efficacy	Quality score
**[^{18}F]FDG) PET/CT**					
Fuster et al	2011	Localized and advanced refractory (n = 15)	SUVmax	Patients with a PFS ≤ 6 months showed a significantly higher SUVmax at baseline compared to patients with PFS ≥ 6 months, using univariate analysis (p < 0.05).	Low quality 3/10
Miyake et al.	2016	Localized (n = 46)	Uptake pattern	Presence of ring-shaped uptake was an adverse prognostic factor for postoperative recurrence (p = 0.015).	Low quality 3/10
Albano et al.	2019	Localized (n = 35)	MTV and TLG	Two tailed log rank test confirmed MTV and TLG as prognostic factors for PFS (p = 0.032, p = 0.045).	High quality 6/10
Hwang et al	2021	Localized (n = 62)	MTV and TLG	MTV and TLG are independent prognostic factors for RFS (p = 0.009 and p = 0.008).	High quality 5/10
**CE-CT imaging**					
O'Neill et al.	2016	Localized and advanced treatment-naïve gastric (n = 143)	Tumor size, shape and enhancement pattern	Tumor size > 10 cm, irregular borders and enhancing solid component are independent predictors of OS with 40 months follow-up (p < 0.001, p = 0.002 and p = 0.003).	High quality 5/10
Chen et al.	2019	Localized (n = 147)	Deep learning	ResNet model predicting 3-year and 5-year RFS with an AUC of 0.912 and 0.887, respectively. Better predictive capability compared to golden standards.	High quality 18/26
Chen et al.	2019	Localized gastric (n = 50)	Invasion	Adjacent tissue invasion is an Independent risk factor for tumor recurrence (p = 0.036).	Low quality 4/10
Ekert et al.	2019	Advanced (n = 25)	Four features (texture)	Combining features to predict disease progression with an AUC of 0.827.	Low quality 5/10
Xu et al.	2020	Localized gastric (n = 155)	Tumor size and shape	Tumor size < 5 cm and smooth defined borders and both indicated higher PFS. (p = 0.005, p < 0.001 and p = 0.023).	Low quality 3/10
Cannella et al.	2021	Localized and advanced (n = 88)	Tumor shape	Ill-defined borders are associated with shorter PFS (p = 0.004).	Low quality 3/10
Ao et al.	2021	Localized (n = 236)	Four features (shape and texture) + 9 clinical features	AUC 0.937, accuracy 88.7%, sensitivity 87.5% and specificity 88.9%in the validation cohort to predict recurrence after 1 year follow-up.	High quality 10/26
Jung et al.	2022	Localized (n = 113)	Tumor location, shape and feeding vessels	Nomogram using tumor location (gastric vs. non-gastric), ill-defined borders and presence of feeding vessels showed an AUC of 0.863.	High quality 6/10
Zheng et al.	2021	Localized and advanced (n = 204)	Nine features (shape and texture) + 4 clinical features	Combined model predicted the development of liver metastasis in high risk GISTs with an AUC of 0.873 and 84.9% accuracy, superior to the clinical model.	High quality 16/26

Table 5 Summarized overview of the included articles predicting prognosis, including study characteristics, quality score, evaluated radiological features and their conclusions on efficacy (RFS = recurrence free survival, PFS = progression free survival, MTV = metabolic tumor volume, TLG = total lesion glycolysis and AUC = area under the curve)

Table 6 | **Monitoring of TKI treatment response**

Author	Year	Patient group	TKI treatment	Results on efficacy	Quality score
**[^{18}F]FDG) PET/CT**					
Ryu et al.	2006	Advanced or recurrent (21 patients)	Imatinib	OS was significantly better in patients with focal progression and the appearance of a new cystic lesion, compared to general progression and new solid lesion patterns (p = 0.0157).	*Low quality* High risk of bias Concerns for applicability
Phongkitkarun et al.	2008	Advanced or recurrent (17 patients & 62 lesions)	Imatinib	OS was significantly better in patients with cystic change or response determined by RECIST, compared to patients with general progression (p = 0.0271).	*Low quality* Low risk of bias Concerns for applicability
Dudeck et al.	2010	Advanced with progression under imatinib (54 patients & 176 lesions)	Sunitinib	SD according to RECIST displayed similar PFS compared to PR and SD determined by Choi criteria at 3 months follow-up (p = 0.684 and p = 0.690).	*Low quality* High risk of bias No concerns for applicability
Schiavon et al.	2012	Advanced liver metastases (84 patients)	Imatinib	Volumetric measurements detected a size change of ≥ 20% in a higher number of liver metastases, compared to RECIST.	*High quality* No risk of bias Low-level concerns for applicability
Schramm et al.	2013	Advanced with progression under imatinib (20 patients & 68 lesions)	Sunitinib	Comparable results in DSS between RECIST and Choi after 3 month follow-up. Only PR determined by RECIST indicated favorable survival outcomes after 1 year follow-up.	*High quality* Low risk of bias Low-level concerns for applicability
Shinagare et al.	2014	Advanced with progression under imatinib and sunitinib (n = 62)	Regorafenib	RECIST performed better with a median PFS of 35 weeks, compared to Choi criteria with a 23 weeks PFS after one year follow-up.	*High quality* Low risk of bias Low-level concerns for applicability
Schiavon et al.	2014	Advanced liver metastases (78 patients & 139 lesions)	Imatinib	The volume determined by RECIST criteria overestimated the actual volume of liver metastases with 35.0%.	*High quality* No risk of bias Low-level concerns for applicability
**CE-CT imaging**					
Goh et al.	2006	Localized and recurrent advanced	Imatinib	Poor correlation between pathological complete response and FDG-PET findings (18/37 vs. 3/37).	*Low quality* High risk of bias Concerns for applicability
Prior et al.	2009	Advanced with progression under imatinib (n = 23)	Sunitinib	Early FDG-PET metabolic response was significantly associated with prolonged PFS (p = 0.046) compared to stable or progressive disease (p < 0.001).	*High quality* Low risk of bias No concerns for applicability
Chacón et al.	2015	Advanced with progression under 400 mg imatinib (16 patients & 96 lesions)	Imatinib	Early metabolic response determined by EORTC PET criteria on FDG-PET/CT imaging at 7 days was not correlated with PFS.	*High quality* Low risk of bias No concerns for applicability
Schindler et al.	2016	Advanced with progression under imatinib (66 patients & 176 lesions)	Sunitinib	Pharmacokinetic model simulating metabolic activity of GISTs over the course of sunitinib treatment and showed early FDG-PET response to be predictive for OS (HR = 0.16).	*Low quality* High risk of bias Concerns for applicability
Farag et al.	2018	Localized (63 patients and 70 scans)	Imatinib	FDG-PET imaging changed management in 27.1%, which was correlated with a lack of metabolic response (P < 0.001).	*High quality* Low risk of bias Low-level concerns for applicability
Farag et al.	2021	Advanced (39 patients and 61 scans)	Imatinib, sunitinib, regorafenib and nilotinib	Early FDG-PET imaging changed management in 5.6% of patients, while late imaging outcomes changed management in 56.0%.	*High quality* Low risk of bias Low-level concerns for applicability
**[18F]FDG) PET/CT vs. CE-CT imaging**					
Stroobants et al.	2003	Advanced (17 patients)	Imatinib	There was an agreement between RECIST and EORTC PET criteria in 85.7%. One-year PFS was significantly higher in PET responders (SD and CR) compared to non-responders (p = 0.0012).	*High quality* Low risk of bias Low-level concerns for applicability
Jager et al.	2004	Advanced (14 patients)	Imatinib	The mean reduction of SUVmax was significantly higher in good RECIST responders (p = 0.002) and early FDG-PET response was associated with a longer PFS with (p = 0.002).	*High quality* Low risk of bias Low-level concerns for applicability
Antoch et al.	2004	Advanced (20 patients & 282 lesions)	Imatinib	Accuracy of early EORTC PET criteria on FDG-PET/CT was 95.0% after one month and 100% for the 3 and 6 months follow-up.	*High quality* No risk of bias Low-level concerns for applicability
Choi et al.	2004	Advanced (36 patients & 173 lesions)	Imatinib	SD was documented in 75.0% of patients using RECIST, while 70% showed SUVmax reductions between 61-100% after 2 months.	*High quality* Low risk of bias Low-level concerns for applicability
Gayed et al.	2004	Advanced and recurrent (54 patients)	Imatinib	FDG-PET/CT predicted EORTC PET response criteria two months earlier in 22.5% of the patients when compared to RECIST on CE-CT.	*Low quality* High risk of bias Low-level concerns for applicability
Goerres et al.	2004	Localized and advanced (28 patients)	Imatinib	Loss of FDG accumulation on post-treatment FDG-PET scans was significantly associated with longer PFS (p = 0.002).	*Low quality* High risk of bias Low-level concerns for applicability

Table 6 Summarized overview of the response monitoring of various TKI treatments, including study characteristics, quality score, evaluated radiological features and their conclusions on efficacy (OS = overall survival, DSS = disease specific survival, HR = hazard ratio, PFS = progression free survival, SD = stable disease, RECIST = response evaluation criteria in solid tumors and EORTC = european organization for research and treatment in cancer)

Table 6 | Monitoring of TKI treatment response

Author	Year	Patient group	TKI treatment	Results on efficacy	Quality score
**[18F]FDG) PET/CT vs. CE-CT imaging**					
Beheshti et al.	2007	Localized and advanced (15 patients & 67 lesions)	Imatinib	FDG-PET/CT predicted EORTC PET response criteria earlier in 18.0% of the lesions when compared to RECIST criteria on CE-CT.	*High quality*<br>No risk of bias<br>Low-level concerns for applicability
Choi et al.	2007	Advanced (40 patients & 172 lesions)	Imatinib	Choi criteria in line with EORTC PET criteria with sensitivity and specificity of 97.0% and 100% versus 52.0% and 100% for RECIST response.	*High quality*<br>Low risk of bias<br>Low-level concerns for applicability
Holdsworth et al.	2007	Advanced (58 patients)	Imatinib	New thresholds showed significant split between two populations in TTF (both $p < 0.0001$) and outperformed both RECIST and EORTC PET criteria.	*Low quality*<br>High risk of bias<br>Concerns for applicability
Van den Abbeele, et al.	2012	Localized and advanced (57 patients)	Imatinib	Significant reduction in SUVmax after 1 week of treatment ($p < 0.001$). Partial response was determined in 81.8% of patients using EORTC PET criteria and only 5.13% using RECIST.	*High quality*<br>No risk of bias<br>Low-level concerns for applicability

Table 6 Summarzsed overview of the response monitoring of various TKI treatments, including study characteristics, quality score, evaluated radiological features and their conclusions on efficacy (TTF = time-to-treatment failure, SUV = standardized uptake value, RECIST = response evaluation criteria in solid tumors and EORTC = european organization for research and treatment in cancer)
